# Porous Iridium Oxide Inverse Opal Catalysts Enable Efficient PEM Water Electrolysis

**DOI:** 10.1002/adma.202514659

**Published:** 2026-01-06

**Authors:** Sebastian Möhle, Kerolus Nasser Nagi Nasralla, Jakub Drnec, William Chèvremont, Peter Strasser

**Affiliations:** ^1^ Department of Chemistry Technical University Berlin Straße des 17. Juni 124 10623 Berlin Germany; ^2^ ESRF–The European Synchrotron Grenoble 38043 France

**Keywords:** electrolysis, Inverse Opals, iridium, OER, PEM‐WE

## Abstract

Green hydrogen produced by proton exchange membrane water electrolysis (PEM‐WE), has gained significant attention as a future energy carrier and as a feedstock for the chemical industry. Reducing the use of scarce iridium in PEM‐WE anodes is a critical requirement. In this work, porous iridium‐based inverse opal structures (**IrO_x_‐IO**) of varying pore sizes are introduced as novel unsupported bulk anode catalysts and their superior performance compared to commercial alternatives is demonstrated. The influence of porosity and surface area on the electrochemical performance is systematically investigated and categorized using voltage breakdown analysis and equivalent circuit modeling. Efficient **IrO_x_‐IO** operation requires balancing surface area and pore size, enabling high performance up to 13 A cm^−^^2^ with iridium utilizations below 0.1 g_Ir_/kW at 70% efficiency. The findings advance our understanding of unsupported bulk catalysts and, more importantly, expand the range of viable anode materials by clarifying how catalyst morphology influences electrode reactivity.

## Graphical abstract

Porous iridium‐based inverse opal (IrOx‐IO) structures are introduced as high‐performance, unsupported PEM‐WE anode catalysts. Their electrochemical behavior is analyzed through porosity/surface area tuning, voltage breakdown, and circuit modeling. IrOx‐IO anodes reach 13 A cm^−^^2^ at <0.1 gIr/kW with structural stability, offering enhanced iridium utilization and insights into morphology–performance relationships for future green hydrogen production.

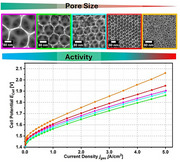

## 1 Introduction

The use of water electrolysis has gained significant attention as a potential replacement for conventional hydrogen production from fossil sources.^[^ ^1^ ^]^ In particular, acidic water electrolysis employing a proton exchange membrane (PEM) as a solid electrolyte is a relatively mature and already commercialized technology.^[^ ^2^ ^]^ Nevertheless, some drawbacks remain. Due to the harsh operating conditions – high potentials combined with a low pH at high temperatures – corrosion‐resistant materials must be used for both cell hardware and electrocatalysts.^[^ ^3^, ^4^ ^]^ In particular, the rare and expensive metal Iridium, commonly used for the anodic oxygen evolution reaction, is a critical material. To make PEM water electrolysis (PEM‐WE) more cost‐efficient, especially regarding upscaling to the MW scale, the total geometric Ir loading must be significantly reduced from ≈2 mg cm^−^^2^ to below 0.5 mg cm^−^^2^.

This can be achieved, for example, by supporting Iridium on a corrosion‐resistant material such as TiO_2_ or by combining it with other metals or elements.^[^ ^5^, ^6^, ^7^, ^8^ ^]^ Another approach involves preparing Iridium‐based bulk catalysts with controlled morphologies, such as nanoparticles or highly porous bulk materials.^[^ ^9^, ^10^, ^11^, ^12^ ^]^ This strategy relies on achieving a high surface‐to‐volume ratio for optimal utilization of iridium atoms. This may, for example, be achieved through Iridium nanoparticles, as numerous studies have demonstrated. While ultra‐high surface areas of up to 150 m^2^ g^−1^ have been obtained using IrO_2_ nanoparticles synthesized via the Adams fusion method, the size and porosity of particle agglomerates certainly play a critical role.^[^ ^13^ ^]^ Especially at the cell level – where catalyst performance depends on more than just physical and chemical properties, such as crystallinity and electrochemically active surface area (ECSA) – other material characteristics must also be considered:^[^ ^14^ ^]^

Bernt et al. found that a low packing density is important for the preparation of high‐performance catalyst layers.^[^ ^15^ ^]^ The conductivity of the catalyst materials must be sufficiently high to reduce the overall cell voltage.^[^ ^16^ ^]^ The ionomer must be evenly distributed throughout the cross‐section of the catalyst layer to provide the crucial triple‐phase boundaries – consisting of electrolyte, ionomer, and catalyst – and thus optimize catalyst utilization.^[^ ^17^ ^]^ Finally, rapid diffusion of electrolyte to, and products away from, the catalytically active centers must be ensured to minimize losses from potentially avoidable mass transport overpotential. However, the influence of mass transport phenomena in the context of PEM‐WE is difficult to explore, especially with regard to the anode catalyst layer.

Because mass transport limitations in PEM‐WE are less apparent due to the absence of a distinct voltage increase, meticulous voltage breakdown analyses are necessary to deconvolute the various contributions to the overall cell potential.^[^ ^18^ ^]^ Although several studies exist, they typically focus on cell hardware (e.g., the flow field), gas evolution and bubble formation, or the porous transport layer on the anode side of the PEM electrolyzer.^[^ ^19^, ^20^, ^21^, ^22^, ^23^, ^24^ ^]^

To this date, few studies have explicitely examined the influence of anode layer properties on mass transport. Most research has focused on the implications of ionomer content and its effect on catalyst layer thickness.^[^ ^17^, ^25^, ^26^, ^27^ ^]^ Some studies have investigated the effect of anode catalyst layer porosity by introducing pores through the addition of a porogen, but an in‐depth analysis of the porosity of the employed catalyst particles and the implications on the electrochemical performance in a electrolyzer environment is still pending.^[^ ^28^, ^29^ ^]^

In this study, we explore a family of iridium oxide‐based inverse opals (**IrO_x_‐IO**) with tailored pore size distributions and high surface areas. Physical characterization was conducted to assess how pore size influences electrochemical performance in a single‐cell environment. We demonstrated that an optimal pore size range exists, balancing mass transport losses caused by uneven ionomer distribution. A direct comparison revealed that our best‐performing material achieves superior performance at an anode loading 2‐ to 4‐times lower loading. A short‐term durability measurement and catalyst layer analysis confirmed the retention of the inverse opal morphology.

## 2 Synthesis and Characterization of Ir‐Based Inverse Opals

**Figure 1:**
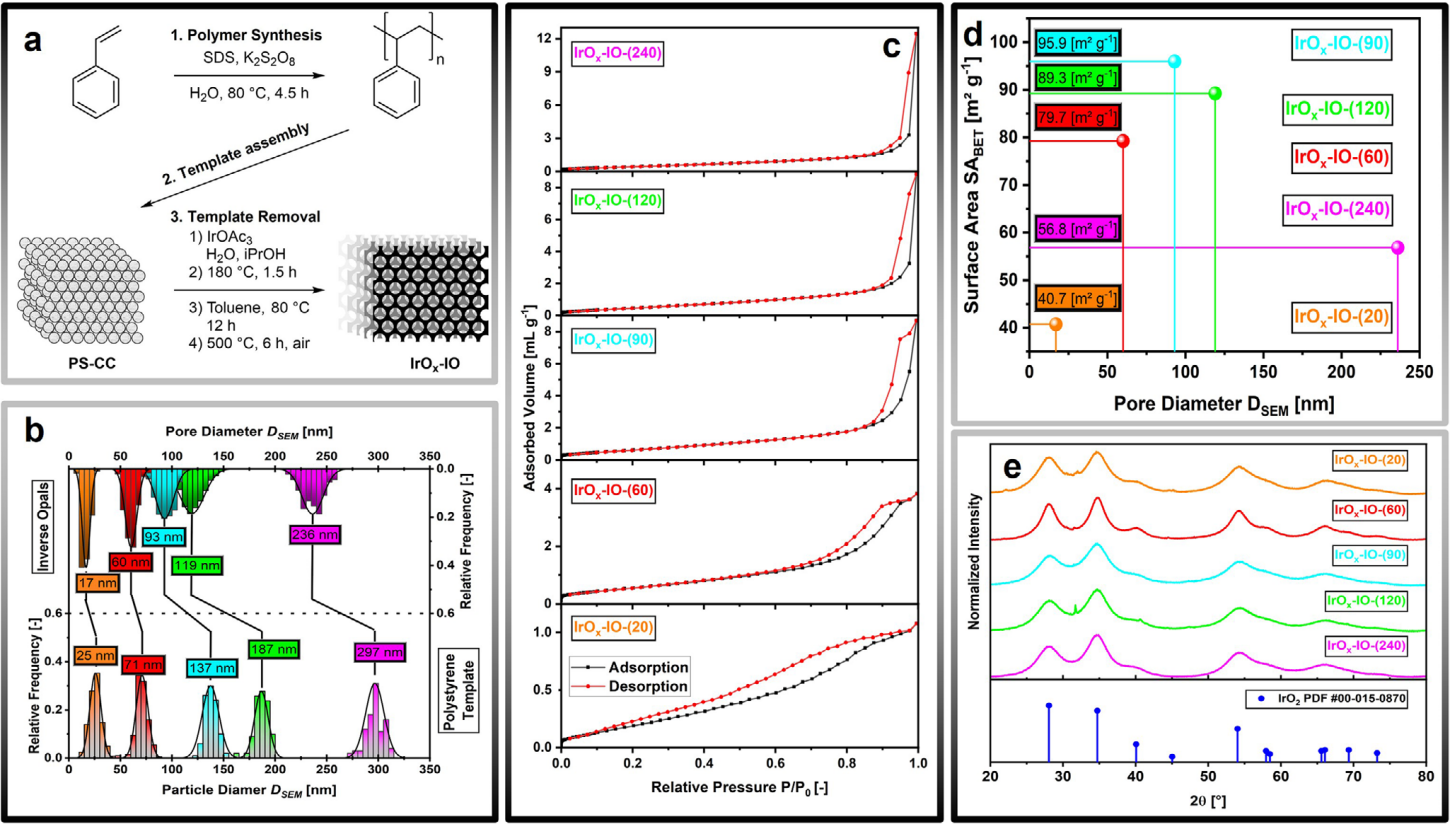
Synthetic procedure and physical characterization of both polymer template and inverse opal catalysts. a) Synthesis protocol for the preparation of polystyrene colloidal crystal (**PS‐CC**) and iridium oxide inverse opals (**IrO_x_‐IO**). b) Comparison of particle and pore diameter of both template and the inverse opal catalysts. c) N_2_‐physisorption isotherms consisting of adsorption (black) and desorption (red) branches of the inverse opal catalysts of varying pore sizes. d) Correlation plot of the surface area determined by the BET‐isotherm against the SEM pore size. e) Normalized powder X‐Ray diffractograms of the synthesized IrO_x_ catalysts of varying pore sizes.

The synthesis procedure is illustrated in **Figure** **1**a. The polystyrene microspheres, which serve as the template, were synthesized via conventional radical emulsion polymerization.^[^ ^30^, ^31^ ^]^ Size control of the microspheres was achieved by adjusting the surfactant concentration, as shown in Figure S1a (Supporting Information). The resulting aqueous polymer dispersion is assembled into a colloidal crystal (**PS‐CC**) by gently drying the polymer suspension at room temperature. Next, the solidified polymer is infiltrated with an iridium acetate solution. The impregnated template is briefly heated and then leached with toluene before annealing. To ensure both the full oxidation of the iridium precursor and the complete removal of the polymer template, a maximum temperature of 500°C was chosen.

**Figure 2:**
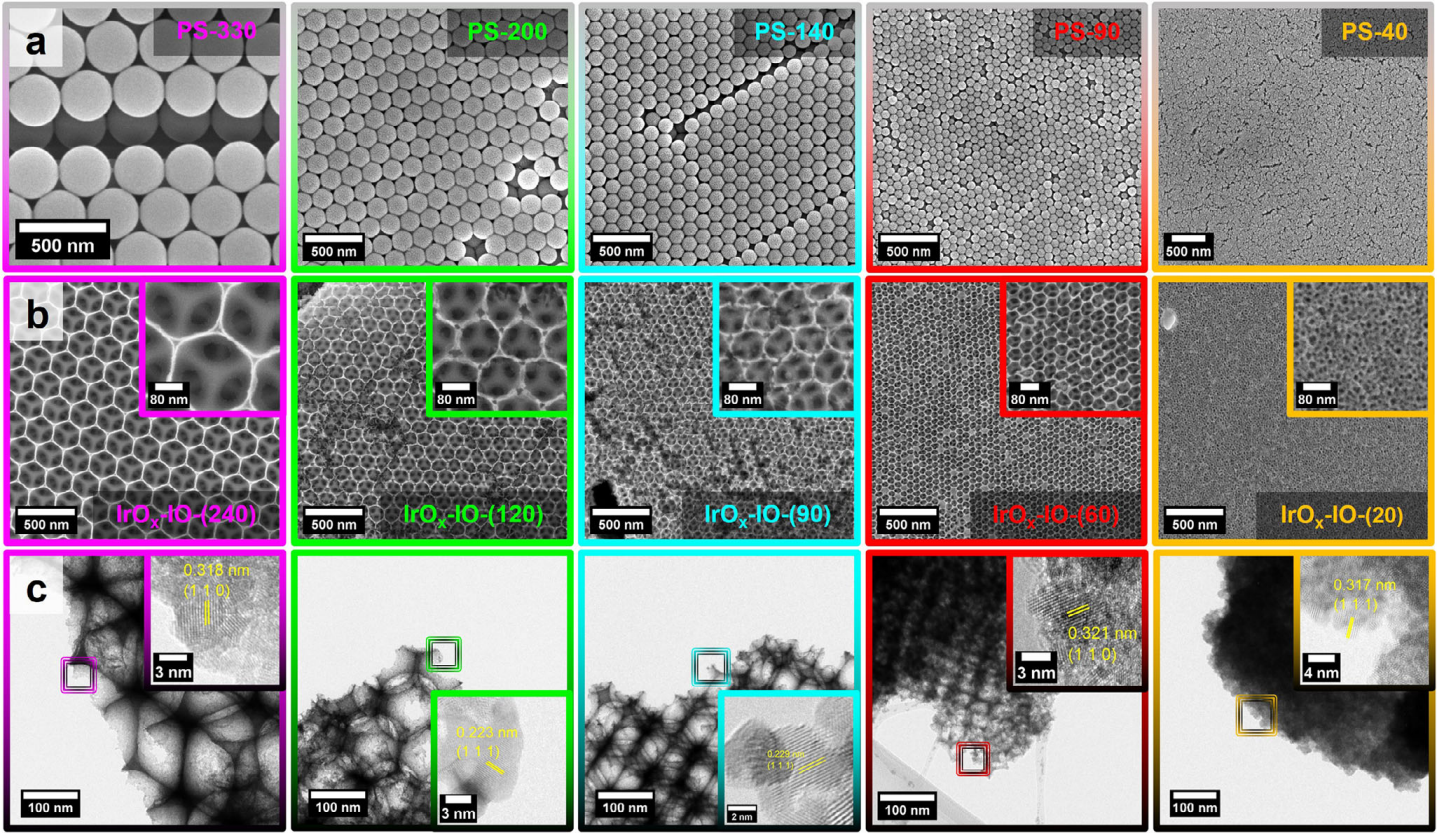
Electron micrographs of the synthesized polystyrene templates and the IrO_x_‐IO. a) SEM‐micrographs of polystyrene template assembled in a colloidal crystal. b) SEM micrographs of porous iridium oxide catalysts. Inserts: Zoom‐in view. c) TEM micrographs of the porous iridium oxide catalysts. Inserts: High resolution micrographs.

A direct comparison of the polymer diameter of the template and the pore diameter of the **IrO_x_‐IOs**, as determined by scanning electron microscopy (SEM), is shown in Figure 1b. Both pore and particle size distributions follow a normal distribution. A shrinkage of 30–40% of the pore size with respect to the corresponding template size is observed, which is consistent with commonly reported values.^[^ ^32^ ^]^ Scanning respectively transmission electron micrographs (TEM) of both polystyrene and the iridium catalyst are shown in **Figure** **2**. For templates **PS‐330** to **PS‐90**, a highly ordered colloidal crystal structure is observed. The materials **PS‐40** and **IrO_x_‐IO‐(20)**, on the other hand, do not exhibit significant long‐range order and show a broad particle and pore size distribution (Figures S3f–S4f, Supporting Information), indicating the presence of a colloidal amorphous array in the case of **PS‐40**.^[^ ^33^, ^34^ ^]^

We conducted small‐angle X‐ray scattering (SAXS) measurements that not only support the existence of long‐range order observed by electron microscopy but were also used to determine the particle and pore sizes of the prepared materials (Figures S5–S8, Supporting Information). As shown in Figure S5 (Supporting Information) for **PS‐90**, our laboratory instrument produced a scattering pattern displaying distinct Bragg spots. Since polystyrene microspheres with a narrow size distribution crystallize in an *fcc* lattice, these diffraction spots can be assigned to specific crystallographic planes.^[^ ^34^, ^35^ ^]^ From the d‐spacing, calculated based on the positions of the reflections and the known *hkl* values, the diameters of the spheres as well as the pores can be determined. As expected from the micrographs of **PS‐40** and **IrO_x_‐IO‐(20)**, the scattering curves exhibit only broad and ill‐defined features. Due to the lower degree of ordering of the polystyrene spheres, the calculation of particle and pore size cannot be performed in the same manner as for **PS‐90**. Similar experiments on the templates **PS‐330** to **PS‐140** and their corresponding inverse opals were conducted at a synchrotron facility, as smaller scattering angles are required for larger spheres and pores. The scattering patterns are shown in Figure S7 (Supporting Information), confirming the long‐range order of the synthesized materials. Furthermore, the sphere and pore diameters calculated from the diffraction peaks match the hydrodynamic diameter of the template, as well as the size distributions determined by electron microscopy (Figure S8, Supporting Information). A more exhaustive analysis of the SAXS experiments can be found in the supplementary information (Note S2, Supporting Information).

To investigate the porosity and surface area of our materials, we performed N_2_‐physisorption experiments. The materials **IrO_x_‐IO‐(240)** and **IrO_x_‐IO‐(120)** both follow the type II adsorption isotherm commonly observed for macroporous solids.^[^ ^36^ ^]^ A weak, H3 hysteresis loop in the desorption branch is observed, which becomes more pronounced with decreasing pore size (**IrO_x_‐IO‐(120)**). The adsorption isotherm of **IrO_x_‐IO‐(90)** still follows the type II isotherm, while a more pronounced hysteresis in the desorption branch further indicates a higher degree of mesoporosity. The pronounced kink clearly marks the transition from the macroporous to the mesoporous domain. The adsorption isotherm of **IrO_x_‐IO‐(60)** also exhibits this transition, as indicated by the kink at high pressures. A clear assignment to the commonly observed isotherms cannot be made. Instead, it appears to be a mixture of type II and IV isotherms. This is supported by both the previously discussed micrographs and scattering patterns. Furthermore, a strong hysteresis, typically observed in mesoporous materials, is present. Finally, **IrO_x_‐IO‐(20)** appears to consist primarily of mesopores, with a possible presence of micropores, as indicated by the low‐pressure regime of the adsorption isotherm. The pronounced hysteresis in the desorption branch indicates the predominance of mesopores, supporting our previous analysis by SEM, TEM and SAXS.

In Figure 1d, the BET surface area is plotted against the pore diameter measured by SEM. As expected from our theoretical considerations found in Note S2 (Supporting Information), the surface area strongly increases with decreasing pore diameter, reaching a maximum of 95.9 m^2^ g^−1^ at a pore size of ≈ 90 nm. Beyond this point, the surface area decreases significantly to 40.7 m^2^ g^−1^ at a pore diameter of 17 nm. The discrepancy between the surface areas of the pristine templates (Figure S1c, Supporting Information) and the porous materials suggests potential limitations of the preparation method used in this study. This is further supported by the scarcity of literature studies utilizing ultrasmall polymer nanospheres for templated synthesis, as well as the preference for the soft‐templating approach using surfactants for the preparation of mesoporous materials.^[^ ^37^ ^]^

To determine the crystal structure of our materials, we conducted X‐ray diffraction (XRD) experiments (Figure 1e). The comparison with the IrO_2_ reference clearly proves the presence of the rutile crystal structure, which was confirmed using high‐resolution transmission electron microscopy (HR‐TEM, Figure 2c). The d‐spacing determined from atomic‐resolution images strongly implies the existence of rutile IrO_2_. Broad diffraction reflections in Figure 1e indicate small crystallite sizes. As this study investigates the influence of both pore size and surface area, it is crucial to minimize any effects on electrochemical activity and electrical conductivity resulting from potentially significant discrepancies in the grain sizes of the investigated materials. Therefore, we performed Le Bail fitting to determine the average crystallite sizes (Figure S9, Supporting Information). Our analysis shows that the crystallite sizes lie between 2.0 and 3.6 nm (Table S3, Supporting Information). Although a precise determination of the grain size distribution by electron microscopy is challenging, the crystallites observable in the TEM micrographs (Figure 2c) confirm the results from whole‐pattern fitting. No clear correlation between grain size and pore size can be observed as shown in Table S3 (Supporting Information).

## 3 Inverse Opals Boosting Performance for Efficient Water Electrolysis

**Figure 3:**
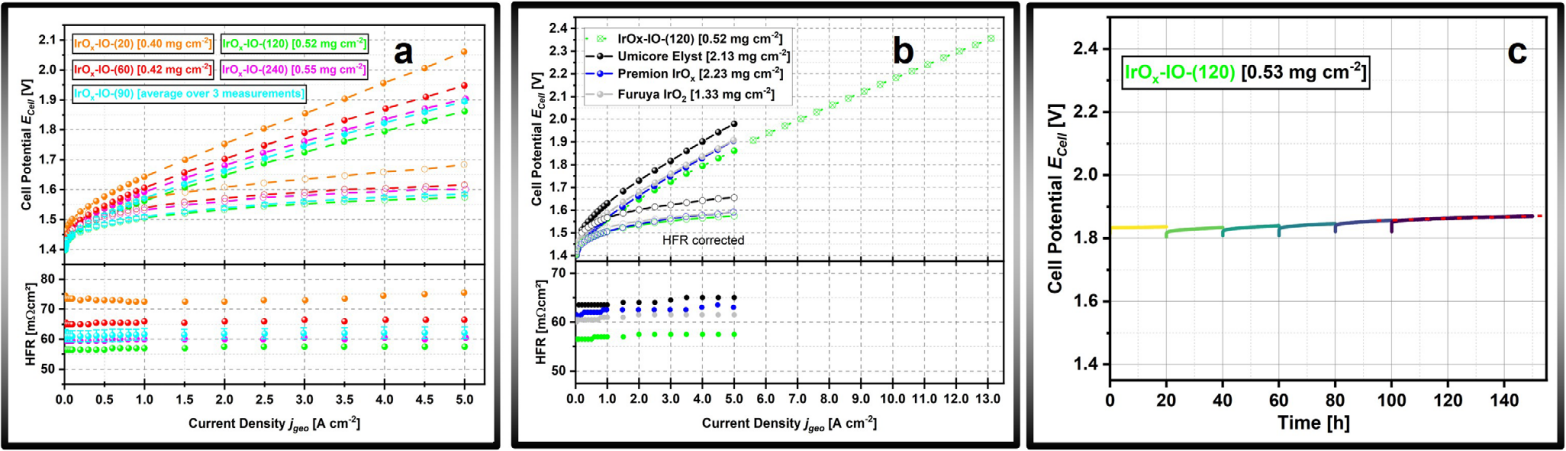
Performance testing of iridium oxide‐based inverse opals in a PEM water electrolyzer. a) Galvanostatic PEM‐WE polarization curves of IO anodes (top) along with the corresponding high frequency cell resistances (bottom). b) Comparison of cell performace of **IrO_x_‐IO‐(120)** (green) with *state‐of‐the‐art* commercial benchmark catalysts; performance test of **IrO_x_‐IO‐(120)** up to ultrahigh current densities. c) Stability performance test of **IrO_x_‐IO‐(120)** using a Nafion 115 membrane at 2 A cm^−^^2^. Test conditions: Both cathode (Umicore Elyst 50, 0.11 mg_Pt_ cm^−2^) and the iridium catalyst were coated onto the membrane (Nafion NR 212, 5 cm^2^) using the decal transfer process. Carbon paper (Sigracet 22 BB) was used as the gas diffusion layer on the cathode side, while Pt‐sputtered titanium felt was used as porous transport layer on the anode side. The applied torque was 35 Nm. An anode flow of 50 mL min^−1^ (100 mL min^−1^ for ultrahigh current densities) at 1 atm pressure and 80°C was used. For ultrahigh current densities an unheated cathode flow of 100 mL min^−1^ was used to remove heat from the MEA.

The measured polarization curves, along with the corresponding geometric mass loading, are shown in **Figure** **3**a. To provide a rough estimate of the measurement uncertainty, three MEAs composed of **IrO_x_‐IO‐(90)** were prepared and analyzed. The average current densities from three separate measurements, along with their standard deviations, are shown in Figure 3a. A more detailed discussion of the reproducibility is provided in the supplementary information (Note S3, Supporting Information). At each current step, the high‐frequency resistance (HFR) was determined by the intersection of the recorded impedance data with the real component of the cell impedance. The corresponding HFR‐corrected polarization curves are plotted using hollow symbols. For a clearer overview of the exact data, the measured and HFR‐corrected cell potentials at a geometric current density of 5 A cm^−^^2^, where mass transport effects are expected to be most significant, are tabulated in Table S3 (Supporting Information). Across all curves, **IrO_x_‐IO‐(120)** exhibits the lowest overpotential, while **IrO_x_‐IO‐(90)** demonstrates a marginally better performance than **IrO_x_‐IO‐(240)**, though within the margin of error. **IrO_x_‐IO‐(60)** performs slightly worse, exhibiting an overpotential 86 mV higher (*E_HFR_* 42 mV) than **IrO_x_‐IO‐(120)**. Finally, **IrO_x_‐IO‐(20)** shows the lowest performance among our materials, with an overpotential ≈230 mV (*E_HFR_* 109 mV) higher than **IrO_x_‐IO‐(120)**. At high current densities, a slight kink can be observed, indicating the onset of mass transport limitations. The HF resistances shown in both Table S3 (Supporting Information) and Figure 3a reveal a significantly higher value of ≈65 mΩcm^2^ respectively 77 mΩcm^2^ for **IrO_x_‐IO‐(60)** respectively **IrO_x_‐IO‐(20)**. All other materials exhibit comparable HFRs within the margin of error.

We furthermore measured three different commercially available benchmark catalysts to demonstrate the excellent performance of our **IrO_x_‐IO**s (Figure 3b). At drastically lower loading of 0.52 mg cm^−^^2^ our **IrO_x_‐IO‐(120)** shows significant lower overpotential than iridium oxide (*Umicore Elyst75* IrO_2_/TiO_2_: 2.13 mg cm^−^^2^, 1.98 V at 5 A cm^−^^2^; *AlfaAesar Premion* IrO_x_ 2.23 mg cm^−^^2^, 1.91 V at 5 A cm^−^^2^; *Furuya* IrO_2_: 1.33 mg cm^−^^2^, 1.91 V at 5 A cm^−^^2^). The HFR free cell potentials show for iridium oxide catalysts from *Furuya* and *Premion* only slightly higher overpotentials, especially in the high‐current regime. The high frequency resistances also show significant higher values for all reference catalysts (≈ 62–70 mΩcm^2^).

As with the further development in cell‐components, operation at ultrahigh current densities may become possible. However in the recent literature only few studies investigate the cell voltage at current densities above 5 A cm^−^^2^, and the focus usually lies more on the cell operation than on catalyst design.^[^ ^38^, ^39^, ^40^ ^]^ We therefore performed a measurement at ultra‐high current densities up to 13 A cm^−^^2^. The overall cell voltage stayed below 2.4 V and no abrupt voltage increase due to mass transport limitation could be observed. While this cannot be directly pinpointed on the inverse opal morphology it still demonstrates the good performance at low loading, even at unusual high current densities. Due to the utilized power source, no impedance spectra could be recorded.

We furthermore performed a 150‐h stability measurement of **IrO_x_‐IO‐(120)** on a thicker membrane (Nafion 115). After 20, 40, 60, 80 and 150 h of testing additional polarization curves were recorded (Figure S21a, Supporting Information). Over the course of the last 50 h a cell voltage increase of > 250 µV h^−1^ was observed. After the stability test, we analyzed the anode catalyst layer cross section by FIB‐SEM. No visible degradation in the highly porous structure of the inverse opals could be observed (Figure S21b, Supporting Information). Using the focused ion beam, a lamella was cut and analyzed by transmission electron microscopy (Figure S21c, Supporting Information). From the high‐resolution images no changes in the crystallite size were observed, indicating that the voltage increase is not caused by a decomposition or restructuring of the inverse opal morphology on an atomic level. Furthermore, we conducted thin film XRD of the used CCM. No major changes in crystal structure can be observed (Figure S22a, Supporting Information).

The impedance spectra recorded in addition to the polarization curves were analyzed using equivalent circuit fitting (Figure S22b, Supporting Information). As can be seen, the high frequency impedance *R_0_* decreases during the first 20–40 h of constant current operation, which can be attributed to the conditioning of the membrane and catalyst layers. With increasing operation time, however, both the high‐frequency and low‐frequency arcs, associated with the charge‐transfer processes of the oxygen evolution reaction, gradually increase. A similar trend is observed for the double‐layer capacitance determined at the anode, indicating time‐dependent changes in the electrochemical interface during prolonged operation. These changes, along with the observed behavior of the polarization curves at both low and high current densities, suggest the gradual contamination of the ion‐exchange polymers and catalyst layers.

This interpretation is consistent with targeted literature studies that have investigated the impact of an‐ and cationic contaminants on the performance and stability of PEM electrolyzers.^[^ ^41^, ^42^, ^43^ ^]^ Furthermore, the conclusion is supported by a comparison with our previous publication examining an almost identical catalyst system.^[^ ^44^ ^]^

The herein demonstrated stability test, however, does only provide limited insight into the overall stability of this catalyst type. While the overall pore architecture seems to be still intact after a prolonged current hold, more tests have to be performed in order to determine its industrial applicability. Apart from chronopotentiometric measurements of beyond 1000 h, stress tests involving thermal as well as potential cycling, to investigate the structural durability of not only the pore morphology, but also the overall catalyst layer toward dynamic conditions would be required. However, an exhausting investigation lies far outside of the scope of this publication, whose main purpose is the inquiry of the pore morphology of the electrochemical activity.

Both our activity and stability measurements demonstrate the fundamental viability of a morphology‐controlled catalyst design as a platform for ultimately reaching DOE‐level Ir loadings (≈0.125 mg_Ir_ cm^−2^). To achieve such low masses further optimization of catalyst deposition and ionomer distribution is required. In addition, modification of the catalytic material itself, for example by incorporating elements such as Nb, Ti, Sn, Ru, or Ta, may be necessary to enhance activity, stability, and conductivity at ultra‐low loading.

## 4 Classifying IO Performance Using Voltage Breakdown Analysis and Impedance Spectroscopy

Deconvoluting the influence of catalyst properties such as pore size and overall material surface area on electrochemical performance is highly challenging and requires sophisticated, *state‐of‐the‐art* approaches. Two possible approaches to address this problem are voltage breakdown analysis (VBA) and fitting an equivalent circuit to impedance data.^[^ ^21^, ^22^, ^24^, ^26^, ^45^, ^46^, ^47^ ^]^ A short introduction into VBA is provided in Notes S4 (Supporting Information). The impedance data recorded at 1.5 V were analyzed by fitting to a commonly used equivalent circuit (Figure S10e, Supporting Information). For the sake of brevity, we refer to the literature for a comprehensive explanation of the theoretical background of the used model.^[^ ^45^, ^46^, ^48^ ^]^

Furthermore, distribution of relaxation times analysis (DRT), which has gained increasing interest in recent years in the field of PEM water electrolysis, was employed to deconvolute the underlying processes.^[^ ^48^, ^49^, ^50^, ^51^, ^52^ ^]^

**Figure 4:**
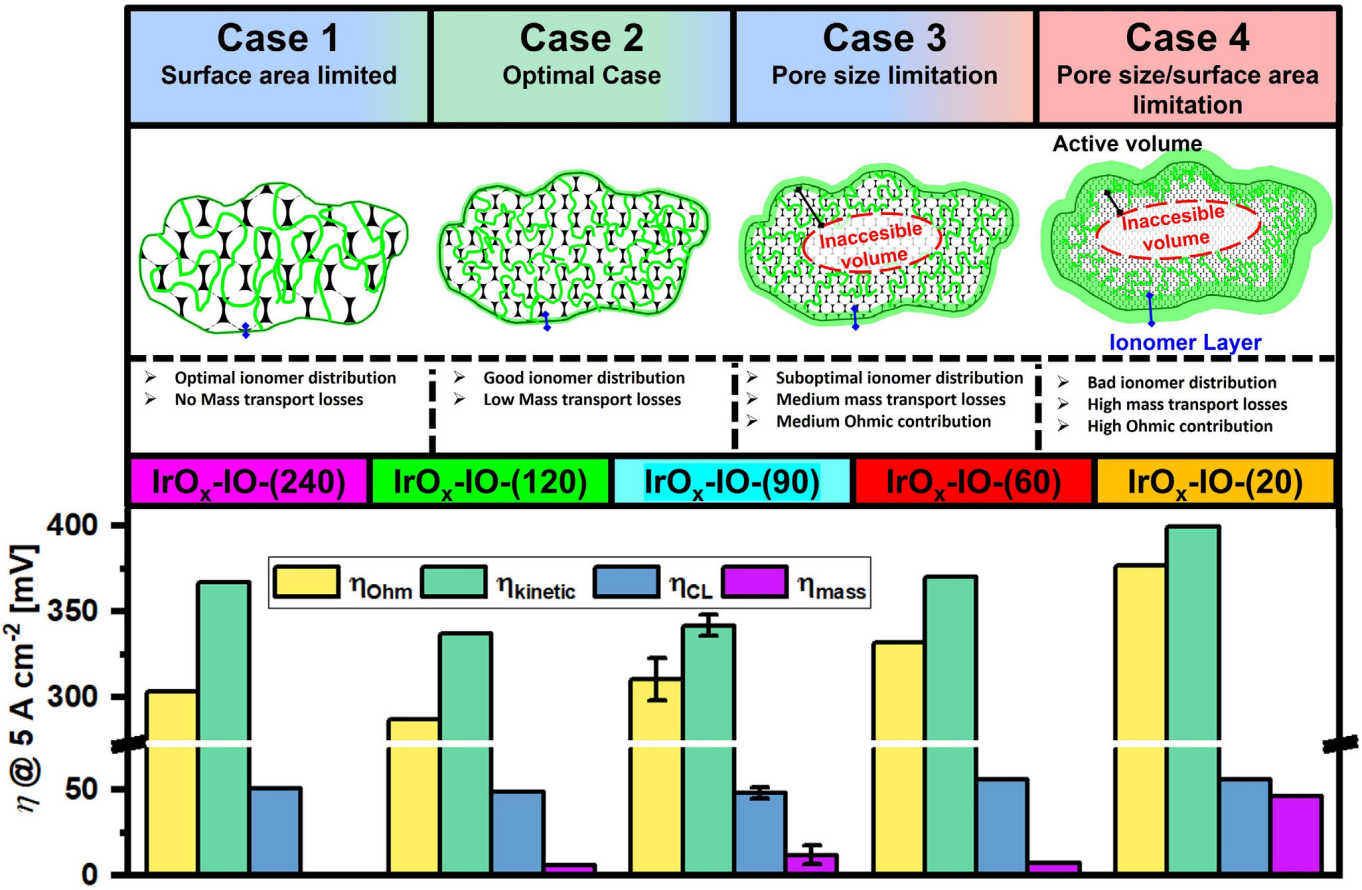
Case distinctions regarding the influence of pore size on the electrochemical performance of Ir‐based inverse opals. *Case 1*: Performance limited by insufficient surface area. *Case 2*: Optimal balance between surface area and pore accessibility. *Case 3*: Performance limited by inadequate pore size for mass transport. *Case 4*: Performance constrained by simultaneous limitations in pore size and surface area. The blue line represents the ionomer layer around the catalyst particles. This layer increases mass transport resistance and partially blocks access to active sites. *Cases 3 and 4*: The black line indicates the catalyst's accessible active volume, where three‐phase boundaries are formed. The red region denotes catalyst volume not covered by ionomer and thus not participating in ionic transport.

The comprehensive evaluation of our experimental data using VBA, equivalent circuit modelling approach and DRT is provided in the Note S5 (Supporting Information). The results of both VBA as well as impedance spectroscopy can be found in Figure S13 (Supporting Information). By combining the results discussed in Note S5 (Supporting Information), we can derive a model for the influence of both pore size and surface area of the catalyst particles on the overall electrochemical performance of our materials (**Figure** **4**). We can distinguish four different cases:

*Case 1 – Surface area limitation*: The surface area‐limited case is best represented by **IrO_x_‐IO‐(240)** and to some extent by **IrO_x_‐IO‐(120)**. Here, the large pore size of the catalyst particles facilitates efficient mass transport both into and out of the material. However, the overall surface area, which can be due to the similarity of the atomic composition and crystal structures used as a proxy for the electrochemical active surface area, remains relatively small and therefore limiting.^[^ ^53^, ^54^ ^]^ The ionomer, crucial for triple‐phase boundary formation, fully penetrates the catalyst particles, ensuring optimal utilization of the available catalyst material. A more uniform ionomer distribution also minimizes additional mass transport resistance caused by the slower diffusion of water or oxygen bubbles through a potentially accumulated polymer film. Uneven coverage of the catalyst particles by the proton‐conducting polymer could block catalytically active sites, increasing the Tafel slope and the kinetic overpotential. Additionally, it might lead to the formation of an electrically insulating film, which would drastically increase the high‐frequency resistance – an effect that is not observed. The slightly higher Tafel slope and kinetic overpotential of **IrO_x_‐IO‐(240)**, compared to **IrO_x_‐IO‐(120)** and **IrO_x_‐IO‐(90)**, indicate that an insufficient number of catalytically active centers are available (site limitation), marking the transition into the surface area‐limited regime. This is further supported by the significantly lower charge transfer resistance and double‐layer capacitance of **IrO_x_‐IO‐(240)** compared to **IrO_x_‐IO‐(120)** and **IrO_x_‐IO‐(90)**.
*Case 2 – Balanced pore size/surface area*: The balanced material property case is best represented by **IrO_x_‐IO‐(120)** and, to some extent, by **IrO_x_‐IO‐(90)**. For these catalysts, both pore size and surface area are well‐balanced. The pores remain large enough to ensure adequate wetting of the internal surface area by the ionomer. The high surface area of the catalyst material is efficiently utilized, resulting in low kinetic overpotential, catalyst‐layer overpotential, and charge transfer resistance. The anodic double‐layer capacitance, determined from impedance spectroscopy, is the highest among all investigated materials. Minor losses due to mass transport (6–12 mV at 5 A cm^−^^2^) are observed.
*Case 3 – Pore size limitation*: The pore‐size‐limited case is best represented by **IrO_x_‐IO‐(60)** and to some extent by **IrO_x_‐IO‐(90)**. While the overall surface area remains high (80–96 m^2^ g^−1^), the pore distribution shifts toward the mesoporous domain (Figure 1c). The ability of the ionomer to penetrate the catalyst particles becomes increasingly difficult, leading to an ionomer‐free core within the catalyst particles. The high surface area cannot be fully utilized, resulting in higher kinetic overpotential and charge transfer resistance, as well as a lower double‐layer capacitance. Since the ionomer cannot fully diffuse into the catalyst particles, it instead forms a film on their exterior, as shown in Figure 4, leading to progressively higher mass transport resistance and lower electric conductivity of the catalyst layer. Uneven ionomer distribution may also block active sites, further deactivating the catalyst. At last, increasing capillary pressure may lead to a stronger binding of oxygen bubbles resulting in gas‐wedge effects inside the pore system, which induce higher mass transport and ohmic resistances.
*Case 4 – Combined surface area and pore limitation*: The surface‐area‐ and pore‐size‐limited case is best represented by **IrO_x_‐IO‐(20)** and, to some extent, by **IrO_x_‐IO‐(60)**. The small pore sizes severely hinder both diffusion into and out of the catalyst particles. Polymer chains, in particular, cannot penetrate the interior of the catalyst particles, further increasing the inaccessible area. As a result, a thick polymer film forms on the exterior of the catalyst particles, significantly impacting electrochemical performance. Active sites in contact with the ionomer become blocked, resulting in a significant increase in kinetic overpotential and charge transfer resistance, as well as a decrease in double‐layer capacitance. Mass transport within the catalyst layer, particularly between catalyst particles, is severely restricted, leading to a high overpotential for the mesoporous materials. Previously mentioned gas‐wedging of trapped oxygen bubbles effect may become more severe with decreasing pore size. Both electrical and ionic conductivity decrease, increasing the catalyst‐layer resistance. Finally, the electrically insulating polymer film increases contact resistance between the PTL and CL, manifesting as higher HF resistance and ohmic overpotential.

While the discussion and model shown in Figure 4 is in agreement with the analysis of our electrochemical data, further experiments are required to fully substantiate these claims. The results and analysis presented in this manuscript provide a solid groundwork for future studies investigating the influence of the pore size of catalyst materials. For a more complete understanding of catalyst–ionomer interactions and active site utilization, future work should not only contain a dedicated ionomer study including the variation of ionomer loading, ink formulation and its chemical structure, but should also employ dedicated model systems rather than full‐cell measurements. For example, the preparation of porous thin‐film electrodes on inert substrates, and their investigation in a three‐electrode configuration, would allow precise determination of turnover frequency and iridium dissolution using techniques such as DEMS and time‐resolved ICP‐MS. The inclusion of a reference electrode would further enable accurate ECSA determination through methods such as mercury underpotential deposition or cyclic voltammetry. Such model systems would also facilitate in‐depth structural and operando analyses, including SAXS or SANS, to elucidate ionomer distribution and bubble‐formation mechanisms during the oxygen evolution reaction. While these experiments extend beyond the scope of the present study, they represent a promising direction for future research to quantitatively resolve the underlying fundamental processes proposed in this work.

## 5 Conclusion

In this work we introduce and demonstrate highly structured iridium oxide inverse opals (**IrO_x_‐IO**) as active and durable alternatives to commercial IrO_2_ PEM‐WE anode concepts. The IO microstructure underwent a broad physical characterization, using various methods like SEM, TEM, physisorption, XRD and SAXS. PEM‐WE performance tests reveal previously unreached current densities of up to 13 A cm^−^^2^ at Ir utilization < 0.1 g_Ir_/kW^−1^ at 70% higher heating value and with excellent structural durability. From the PEM‐WE measurements we were able to draw conclusions on the interplay of the pore size and surface area of the catalyst particles on their electrochemical performance. From here we developed a model that may assist the further development of catalyst materials, and identify morphological features necessary for enhancing iridium utilization. We found, that a balance of surface area and pore sizes is necessary to achieve high performing **IO** anode catalysts. Our study demonstrated that **IO**s bulk **IrO_x_** represent a viable new low‐Ir anode catalyst concept for PEM‐WE technology.

## 6 Experimental Section

### Chemicals and Materials

The following chemicals were used: Nafion solution (Aldrich, 5 wt.% in lower aliphatic alcohols, 15‐20 wt.% water; Aldrich, 20 wt.% in lower aliphatic alcohols, 34 wt.% water), iridium acetate (Heraeus, 47.97% Ir content), deionized water (DI, 18 MΩcm), 2‐propanol (*VWR*, HPLC‐grade), styrene (*Thermo Fisher*, 99%, stabilized), sodium dodecyl sulfate (*Roth*, blotting grade), potassium peroxydisulfate (*Roth*, >99%), and toluene (*Honeywell*, p.a.). Platinum on carbon (*Umicore Elyst Pt50 0380*, 50 wt.% platinum on carbon) was used as the cathode material for electrolyzer measurements. As a commercial reference catalyst, iridium oxide supported on titanium dioxide (*Umicore Elyst 75 0480*, 75 wt.% iridium), Furuya IrO_2_ (82.5 wt.% iridium) and *Alfa Aesar Premion* (84.5 wt.%) were used. Additionally, we used the following materials: Nafion (NR‐212, 50.8 µm thick; 115, 127 µm thick) as the ion‐exchange membrane; FEP (50 µm thick) as the decal substrate for the anode; and Kapton foil (75 µm thick) as the decal substrate for the cathode. Titanium felt (PTL, *Bekaert Currento* Ti‐56/250, 250 µm thick, 56% porosity) was used as the porous transfer layer on the anode side. Carbon paper coated with a microporous layer (GDL, *Sigracet* 22 BB, 190 µm thick) was used as the gas diffusion layer on the cathode side. Gaskets made of polyphenylene sulfide (PPS) were used (cathode: 175 µm; anode: 225 µm).

### General Procedures

All wet‐chemical procedures and experiments, including solvent leaching, polymer synthesis, and the leaching of Ti PTL, were conducted using the Automate parallelized reactor system from HEL.

### Synthetic Procedures—Conventional Emulsion Polymerization

According to a reference procedure, a certain amount of surfactant (sodium dodecyl sulfate) was dissolved in 180 mL of deionized water before the reactor was purged with nitrogen for at least 10 min while stirring at 750 rpm.^[^ ^30^ ^]^ The reaction mixture was then heated to 80°C, and the styrene monomer (1.0 eq, 174 mmol, 18.12 g, 20 mL) was added. After 10 min of stirring, an aqueous solution of potassium peroxydisulfate (0.26 mol%, 451 µmol, 122 mg) in water was rapidly added. The reaction mixture was stirred at 80°C for 4.5 h before being cooled to room temperature. The crude product was purified by filtration to remove larger polymer aggregates. Approximately 200 mL of polymer suspension with a density of 1.0 g mL^−1^ was obtained. The total mass concentration was determined by placing 10 mL of the suspension in a pre‐weighed vial and slowly evaporating the solvent, resulting in a final concentration of ≈87–90 mg_solid_ mL^−1^ and a projected yield of ≈ 18 g.

### Monomer Starved Semicontinuous Microemulsion Polymerization

A modified literature procedure was followed.^[^ ^31^ ^]^ First, a surfactant (sodium dodecyl sulfate, 3.60 mmol, 1.04 g, 20 mM) was dissolved in 180 mL of deionized water before the reactor was purged with nitrogen for at least 10 min while stirring at 750 rpm. The reaction mixture was heated to 80°C and stirred for 20 min. Then, the radical initiator (potassium peroxydisulfate, 1.11 mmol, 300 mg), dissolved in 20 mL of water, was rapidly added. Styrene (28.8 mmol, 3.32 mL, 3 g) was added dropwise using a syringe pump (0.1 mL). Subsequently, a larger amount of monomer (67.2 mmol, 7.74 mL, 7 g) was rapidly added over the course of 1 min before heating the reaction mixture to 85°C. The reaction was allowed to proceed for 5 h before being cooled to room temperature. The crude product was purified by filtration to remove larger polymer aggregates.

### Template Assembly

The polymer suspensions were dried at ambient temperature in crystallization dishes, yielding polystyrene colloidal crystals (PS‐CC).

### Template Infiltration

First, a solution of iridium acetate in water and isopropanol (3:1) with a mass concentration of 44.8 mg_Ir_ mL^−1^ was prepared. Polystyrene (13.9 mg_polystyrene_ mg_Ir_^−1^) was placed in a Petri dish before approximately one‐third of the iridium acetate precursor was added dropwise to the polymer. The solid was allowed to sit for ≈30 min before being thoroughly dried at 40°C under reduced pressure in a vacuum oven. This procedure was repeated until the precursor solution was fully consumed, yielding the infiltrated colloidal crystal as a teal solid. The infiltrated colloidal crystal was then heated to 180°C for 1.5 h.

### Thermal/Chemical Pretreatment

The infiltrated colloidal crystal was then heated to 180°C for 1.5 h. After cooling to room temperature, the sintered polymer was placed in a glass reactor, and toluene (≈200 mL) was added. Under gentle stirring (100 rpm), the mixture was heated to 80°C for 12 h. Then, the solvent was decanted. This procedure was repeated once more. The resulting gel‐like material was transferred to a ceramic crucible and dried at room temperature. Annealing was performed using a conventional tube furnace (*Carbolite Gero*, TS1 12/125/400) equipped with the CC‐T1 cascade controller for precise temperature control. The experiments were conducted in a quartz tube (Ø81.6 mm; ID Ø86 mm × 1000 mm). As the gas atmosphere for the annealing procedure, dried and pressurized air was employed. The gas stream flow rate (60 mL min^−1^) was regulated using a conventional rotameter. A slow heating ramp (1°C min^−1^) with four 3‐h holds at 250, 275, and 300 and 500°C was used to ensure full oxidation and reduce reduction of iridium oxide by the decomposing template.

### Physical Characterization

Dynamic light scattering (DLS): The hydrodynamic diameters of the polymer samples were determined using the *Litesizer 500* from *Anton Paar*. The measurements were performed in quartz cells in backscattering mode (175°) at 25°C. The data were evaluated using the cumulant method. The previously filtered polymer suspensions were diluted with deionized water by a factor of 1:10000. Each sample was measured five times.

### Powder X‐Ray Diffraction (XRD)

The *D8 Advance* laboratory device (*Bruker*), employing a copper source (Cu Kα), was used. Angles between 20° and 80° 2θ were recorded with increments of 0.075°. The powder samples were measured on low‐background silicon holders purchased from *Bruker*.

### Scanning Electron Microscopy (SEM) and Sample Preparation

Samples were analyzed using the *Gemini 982* and *Gemini 500* instruments from *Zeiss* equipped with a field emission gun. For high‐resolution micrographs, low acceleration voltages (≈4 – 6 kV), a small aperture (20 µm), and low working distances (6 – 7 mm) were used. Unless otherwise noted, all micrographs were recorded using the in‐lens detector. Polystyrene samples: The polymer suspensions were diluted with deionized water (DI), and ≈50–100 µL were drop‐cast onto a clean silicon wafer. A thin layer of platinum was sputtered onto the samples to enhance conductivity and reduce charging effects. Particle size distributions were determined using *ImageJ*. A population of at least 100 particles was counted. A normal distribution was fitted to the resulting histogram to determine the average particle size and its standard deviation.

### Inverse Opal Samples

The as‐synthesized polymer suspensions were mixed with a specific amount of iridium acetate. The polystyrene‐to‐iridium ratio used for material preparation was maintained. Approximately 50–100 µL of the mixture was drop‐cast onto a silicon wafer and carefully dried before annealing in the tube furnace.

### Cross Section Samples

The Nafion membranes were cut and embedded in epoxy resin. After the polymer was completely dry, the top side was ground and polished. To reduce charging effects, the top side was coated with a thin layer of carbon. Silver lacquer was applied to further enhance the sample's conductivity. Higher acceleration voltages (10 kV), a 60 µm aperture, and greater working distances (≈10 mm) were used in conjunction with a backscattered electron detector.

### Focused Ion Beam/Scanning Electron Microscopy (FIB‐SEM)

For the preparation of the anode layer cross‐section, the FIB‐SEM *HELIOS 600* from *Thermo Fisher* was used. The machine is equipped with a Schottky emitter and a liquid gallium ion source. Low acceleration voltages (2 kV) were applied. After recording cross‐section micrographs, a lamella was prepared for later TEM measurements.

### High Resolution Transmission Electron Microscopy (HR‐TEM) and Sample Preparation

Transmission electron microscopy was performed using the *FEI TITAN 80–300 Berlin Holography Special*, equipped with a high‐brightness Schottky field emitter (X‐FEG) and operated at 300 kV acceleration voltage. Images were recorded using the *GATAN US1000 CCD* camera.

The samples were prepared by suspending a small amount of catalyst powder (<1 mg) via sonication in water. A small volume of the particle suspension (1.5 µL) was deposited onto a carbon‐coated copper grid and carefully dried.

### Transmission Electron Microscopy (TEM) of Cross Section Samples

The lamella prepared via FIB‐SEM was analyzed using the *Tecnai G2 20 S‐Twin* microscope from *FEI*, equipped with a lanthanum hexaboride source and operated at 200 keV. The images were recorded using the *GATAN MS794 P* detector.

### N_2_‐Physisorption Experiments

Physisorption experiments were conducted using the *Autosorb‐iQ automated gas adsorption station* from *Quantachrome*. A certain amount of sample (20–30 mg) was placed in a glass cell and degassed for at least 4 h at room temperature before measurement. A total of 80 and 40 points were recorded for the adsorption and desorption isotherms, respectively, within a pressure range of p/p_0_ = 10^−7^ – 1. Nitrogen was used as the adsorbate. Data analysis was performed using the *QuadraWin* software from *Quantachrome*.

### Small Angle X‐Ray Scattering (SAXS)

Small angle X‐Ray scattering measurements were conducted using the Nanostar laboratory machine (*Bruker*). The sample‐detector distance used was 104 cm. The X‐Ray source was equipped with a copper target. Ultra small angle X‐Ray scattering measurements were performed at the *European Synchrotron Radiation Facility* (*ESRF, Grenoble*). The beam energy was 12.23 keV with a beam size of 80 × 120 µm^2^.

### Electrochemical Characterization by Single Cell Electrolyzer—Preparation of the Membrane Electrode Assembly (MEA)

A catalyst coated membrane configuration (CCM) for both anode and cathode was chosen. For the comparative experiments with the porous materials presented herein, ≈ 10 mg of catalyst powder was dispersed in water and 2‐propanol at a 1:4 solvent ratio, reaching a mass concentration of 1.25 mg mL^−1^. Then, ≈ 30 µL of Nafion solution (5 wt.%) was added, resulting in a total ionomer content of ≈12% relative to the total solids in the suspension. Larger amounts of catalyst powder were used for the reference materials. The catalyst powder suspension was sonicated using a conventional horn sonifier (*Hielscher*) for 30 min while being cooled in an ice bath. The suspension was then spray‐coated onto FEP foil using an automated ultrasonic spray coater (*Sono‐Tek Corporation, NY 12547*). During the spray‐coating process, the substrates were heated to 90°C at a flow rate of 0.25 mL min^−1^. Bar coating was employed for the preparation of the cathodes. The catalyst slurry was prepared using platinum on carbon (*Umicore Elyst50 0380*, 50wt.% Pt), which was diluted with water and 2‐propanol at a 1:9 solvent ratio, reaching an initial mass concentration of 30wt.% relative to the total mixture weight. A solution of Nafion (20wt.%) in water/aliphatic alcohol was then added. The ionomer content relative to the total solids in the slurry was approximately 17wt.%. Zirconium dioxide spheres were added to the mixture, which was then homogenized on a roller mill overnight. The slurry was further diluted with water (1.9 µL mg^−1^_Pt/C_) reaching a final mass concentration both ionomer and catalyst of ≈ 19 wt.% and homogenized on the roller mill for an additional hour. The mixture was then bar‐coated onto a Kapton foil using a commercially available film applicator (*BEVS, BEVS1818H*) and a Meyer rod (*Thiery GmbH*, 400 g weights, 30 mm s^−1^, 20 µm). The coated substrate was dried at room temperature and then cut into 5 cm^2^ pieces using a cutting plotter (*Cricut, Cricut Maker* 3). Both the anode and cathode were simultaneously transferred onto the Nafion membrane using a hydraulic press (CARVER, 3 min, 155°C, 2.4 MPa). The catalyst loadings were determined by weighing the substrates before and after the decal transfer. The cathode achieved an average loading of ≈0.115 ± 0.01 mg_Pt_ cm^−2^. The porous transport layers were prepared from titanium felt. First, 5 cm^2^ squares were cut using a laser and then etched in an aqueous oxalic acid solution (10wt.%) for 1 h at 80°C. The squares were thoroughly washed with water, isopropanol, and water, then dried at 80°C. The squares were then coated with platinum using a sputter coater. The flow fields were prepared in a similar manner. The gas diffusion layers, containing a microporous layer, were punched out using a 5 cm^2^ stamp and used without further treatment.

### Cell Measurements

The measurements were performed using the *E100 electrolysis test bench* (*Greenlight Innovation*). An anode feed of 50 mL min^−1^, at atmospheric pressure, and a temperature of 80°C was used. A potentiostat (*Reference 3000, Gamry*) and booster (*Reference 30k, Gamry*) were used for all polarization curves, impedance spectra, and cyclic voltammograms. For ultra‐high current densities, the internal power supply (*Sorensen*) of the test rig was used. A short galvanostatic current hold at 1 A cm^−^^2^ was used as a break‐in step. The complete measurement consisted of 5 cyclic voltammograms (CV) between 0.05 and 1.30 V, with a scan rate of 50 mV s^−1^. A potentiostatic impedance spectrum (PEIS) at 1.25 and 1.5 V was performed at 10 Hz and 100 kHz with an amplitude of 10 mV. For both the CVs and the PEIS, the cathode was flushed with hydrogen. The polarization curves were recorded in a current range from 0.001 to 5 A cm^−^^2^ respectively 13 A cm^−^^2^. At each step, a galvanostatic impedance spectrum (GEIS) was measured to determine the high‐frequency resistance. The measurement sequence, consisting of the CV, PEIS, and polarization curve, was performed three times in total. The analysis was performed using the data from the third cycle.

The equivalent circuit (EC) modelling was performed using a self‐written python script using the impedance.py package.^[^ ^55^ ^]^ The distribution of relaxation times analysis was performed using the pyDRTtools package.^[^ ^56^, ^57^, ^58^ ^]^ A more detailed description of the data analysis can be found in Note S5 (Supporting Information).

## Conflict of Interest

The authors declare no conflict of interest.

## Author Contributions

S.M. and P.S. had the original idea and designed the project. S.M. synthesized the materials and conducted the XRD, SAXS, SEM, HR‐TEM, Physisorption, MEA measurements, and analysis. K.N.N.N. prepared the MEAs. J.D. and W.C. performed USAXS measurements. S.M. drafted the manuscript, and P.S. contributed to the final version of the manuscript. All authors discussed the results and commented on the manuscript.

## Supporting information



Supporting Information

## Data Availability

The data that support the findings of this study are available from the corresponding author upon reasonable request.
