# Supplementary material for: Porous Iridium Oxide Inverse Opal Catalysts Enable Efficient PEM Water Electrolysis
Source: Adv Mater. 2026 Jan 6;38(11):e14659. doi: 10.1002/adma.202514659 (PMC12921350; doi:10.1002/adma.202514659)
Supplement: Supplementary file 1 — Supporting Information [file ADMA-38-e14659-s001.docx]

Supplementary Information

Porous Iridium Inverse Opal catalysts enable efficient PEM Water Electrolysis

Sebastian Möhle, Kerolus Nasser Nagi Nasralla, Jakub Drnec, William Chèvremont and Peter Strasser*

S. Möhle, K. N. N. Nasralla and P. Strasser*

Department of Chemistry, Technical University Berlin, Straße des 17. Juni 124, 10623 Berlin, Germany.
E-mail: [pstrasser@tu-berlin.de](mailto:pstrasser@tu-berlin.de)

J. Drnec, W. Chèvremont

ESRF – The European Synchrotron, 38043 Grenoble, France.

**Supplementary Notes 1: SAXS analysis of polystyrene/inverse opal materials**

Electron microscopy suffers the well-known constraint of not being a representative method. We therefore performed SAXS experiments to show both long-range ordering and determine the average particle/pore size. Due to its large beam size (e.g. *h* x *v*: 80 x 120 µm² for data shown in Figure S7) larger proportion of sample is probed at once when compared to scanning or transmission electron microscopy. Hence it can be considered more of a bulk method. The sample preparation is similar to the procedure described in the experimental section with the exception that a thin glass slide (135-160 µm) is used instead. A representative diffraction pattern of both **PS-CC** is shown in Figure S5b. Diffraction rings with prominent Bragg spots can be observed, with the latter indicating large domains of high order and an evenly orientation. The marked Bragg spots can be assigned to the set of planes of either {220} or {440}. From the d-spacing determined from the values of the corresponding scattering vector (1) and the assumption of a cubic crystal system the particle and pore size for both **PS-CC** and **IrO_x_-IO** can easily be calculated.

| $d=\frac{4\pi}{q}$ | (1) |
| --- | --- |

The d-spacing can then be transformed into the lattice constant of the *fcc* by reshaping equation (2) to (3).

| $d=\frac{a}{\sqrt{h^{2}+k^{2}+l^{2}}}$ | (2) |
| --- | --- |
| $a=d\sqrt{h^{2}+k^{2}+l^{2}}$ | (3) |

According to Figure S1b the particle radius and therefore the diameter can then be calculated by applying the Pythagorean theorem (4).

| $r=\frac{\sqrt{2a^{2}}}{4}$ | (4) |
| --- | --- |

The particle and pore diameters determined from SAXS are shown in Figures S7-S8 and are in good agreement with the results obtained from SEM and DLS (Table S1).

**Supplementary Notes 2: Theoretical considerations of the polymer diameter on the properties of the porous iridium oxide materials**

As the polymer microspheres assemble into a colloidal crystal in a face-centered cubic (*fcc,* Figure S1b) arrangement upon drying of their suspension, both the theoretical packing density and the surface area can be easily calculated. Since the pore size and therefore the surface area of the iridium-based inverse opal materials directly depends on the template size, a general trend can be predicted using simple geometrical considerations. The lattice constant ***a*** of the *fcc* structure can be calculated by applying the Pythagorean theorem (Figure S1b, (5)).

| $a=\frac{4r}{\sqrt{2}}$ | (5) |
| --- | --- |

Here, ***r*** denotes the radius of the spheres. The packing density *P_UC_* of the unit cell is defined as the ratio of the volume occupied by the spheres *V_Spheres_* within a unit cell of the *fcc* structure to the total volume of the unit cell *V_UC_*, as shown in equation (6).

| $P_{UC}=\frac{V_{Sphere}}{V_{UC}}=0.74$ | (6) |
| --- | --- |

The quantities *V_Spheres_* and *V_UC_* are calculated using equations (7) and (8), respectively.

| $V_{Spheres}={4*\frac{4}{3}\pi r}^{3}$ | (7) |
| --- | --- |
| $V_{UC}=a^{3}=\frac{64r^{3}}{\sqrt{2}^{3}}$ | (8) |

According to equation (6), the packing density is independent of the radius of the spheres. Since the inverse opals are fabricated by infiltrating the void spaces between the polymer spheres followed by their removal, the packing density of the resulting inverse opal materials should likewise be theoretically independent of the pore radius. The total surface area *SA*, expressed in the conventional unit of m² g^-1^, can then be calculated using equation (9).

| $SA=\frac{S_{UC}}{m_{UC}}= \frac{S_{UC}}{P_{UC}*V_{UC}*\rho}=\frac{3}{\rho r}$ | (9) |
| --- | --- |

Here, ***S_UC_*** represents the surface area of the unit cell, approximated as the combined surface area of the four spheres contained within one unit cell. The packing density *P_UC_* (also known as filling ratio) and the material density must be considered: *P_UC_* = 0.74 for the colloidal crystal (*ρ_Polystyrene_* = 1.09 g cm^-^³) and *P_UC_* = 0.26 for the inverse opals (*ρ_IrO2_* = 10.66 g cm^-^³). According to (9), the surface area of the polystyrene colloidal crystal is inversely proportional to the radius of the spheres. The theoretical values corresponding to the templates used in our experiments are shown in Figure S1c. The experimentally determined surface areas from N_2_-physisorption (Figure S2) not only confirm the predicted trend but also closely match the calculated values within reasonable deviations.

**Supplementary Notes 3: Reproducibility of the cell measurements**

To confirm the validity of our data, we performed three independent measurements of the same sample to estimate deviations arising from variations in MEA preparation. The results are presented in Figure S10 and Table S2. Within a loading range of 0.46 to 0.62 mg cm^-^², the overall cell potential at a current density of 5 A cm^-^² ranged from 1.889 to 1.902 V, with an average value of 1.896 ± 0.005 V. Furthermore, we determined the Tafel slope and fitted an equivalent circuit model to the collected data. The linearity and time-invariance required for valid impedance measurements were confirmed for each dataset using the linear Kramers–Kronig method introduced by Schönleber et al. (Figure S10d, S11a).^[1]^ The observed deviations are comparable to those reported in the literature.^[2]^ The high-frequency resistance exhibited only a slight increase with rising current densities, ranging from 59.5 to 64 mΩcm² at 5 A cm^-^², with an average of 62.2 ± 1.9 mΩcm².

**Supplementary Notes 4: Voltage Breakdown Analysis**

The overall cell voltage *E_Cell_* is considered as the sum of the thermodynamic potential (*E_T,p_*), kinetic losses (*η_kinetic_*), ohmic losses (*η_Ohm_*), and additional contributions from mass transport (*η_mass_*) as well as the catalyst layer resistance (*η_CL_*) (10).

| $E_{Cell}=E_{T,p}+\eta_{kinetic}+\eta_{Ohm}+\eta_{CL}+\eta_{mass}$ | (10) |
| --- | --- |

For brevity, we refer the reader to the literature for detailed derivations of the presented relationships.^[3-7]^

*Thermodynamic potential:* The thermodynamic potential, *E_T,p_*, depends solely on temperature and pressure and can be calculated using the Nernst equation (11).

| $E_{T,p}=E_{T,p}^{0}+\frac{RT}{2F}\ln\left[ \frac{a\left( H_{2} \right)\sqrt{a\left( O_{2} \right)}}{a\left( H_{2}O \right)} \right]$ | (11) |
| --- | --- |

When operating at ambient pressure, the temperature dependence of the cell potential can be approximated using equation (12).^[8]^

| $E_{T,p}\approx1.2291V -0.000846\frac{V}{K}\left( T-298.15 K \right)$ | (12) |
| --- | --- |

*Ohmic contributions:* The ohmic losses, denoted as *η_Ohm_*​, are calculated from the high-frequency resistance *R_HFR_* ​ at each current step, according to equation (13).

| $\eta_{ohm}={jR}_{HFR}$ | (13) |
| --- | --- |

The high-frequency resistance includes the resistance of the overall setup (*R_set-up_*), which comprises the endplates, current collectors, and flow fields (14). In the context of the MEA, both the resistance of the PTL (*R_PTL_*​) and the GDL (*R_GDL_*​) also contribute to *R_set-up_*.

| $R_{HFR}=R_{Set Up}+R_{memb}+R_{CR;PTL}$ | (14) |
| --- | --- |

The overall ohmic resistance also includes the membrane resistance (*R_memb_*) and the contact resistance between the PTL and the catalyst layer (*R_CR;PTL_*).^[5, 9]^ Technically, *R_CR;GDL_* should also be considered, but can be neglected due to the relatively high conductivities of both the carbon GDL and the cathode catalyst. Furthermore, the electric conductivity of the employed catalysts, as well as the contact resistance between the catalyst particles have an impact on the ohmic resistance and consecutively on the HFR.

*Kinetic contributions:* Losses due to kinetic overpotential are derived from the Butler–Volmer equation and can be calculated using the Tafel approximation (15).

| $\eta_{kin}=b\log\left( \frac{j}{j_{0}} \right)$ | (15) |
| --- | --- |

Here *b* is the Tafel slope, *j* the current density and *j_0_* the apparent exchange current density.^[10]^ In our study, we consider the kinetics of the hydrogen evolution reaction to be negligible, as is commonly assumed.^[7]^ As a consequence, the kinetic overpotential (*η_kinetic_*) should depend solely on the oxygen evolution reaction and can be calculated from the Tafel slope determined in the current range of 0.01–0.1 A cm^-^², where kinetic losses are dominant.^[11]^

*Catalyst Layer resistance:* The resistance of the catalyst layer (*R_CL_*​), which comprises both ionic and electronic contributions, is derived from the De Levie model of porous electrodes.^[12-13]^ It may be determined either by fitting a transmission line model to the impedance data or by extrapolating the x-axis intercept of the Nyquist plot (Figure S10f-h and S11c-g).^[7, 10]^ The overpotential due to the catalyst layer resistance can then be calculated from the catalyst layer utilization *u(j)*, as suggested by Alia et al. (16).^[7]^

| $u(i)=\left( 1+\left( \frac{jR_{CL}\ln\left( 10 \right)}{2b} \right)^{\alpha} \right)^{-\frac{1}{\alpha}}$ | (16) |
| --- | --- |

Here, a is a scaling parameter with a value of 1.1982. The catalyst layer overpotential can then be calculated using equation (17):

| $\eta_{CL}=-b log\left( u \right)$ | (17) |
| --- | --- |

*Mass Transport:* The mass transport losses can finally be calculated by reshaping equation (10):

| $\eta_{mass}=E_{Cell}-E_{T,p}^{0}-\eta_{kin}-\eta_{Ohm}-\eta_{CL}$ | (18) |
| --- | --- |

The mass transport overpotential (*η_mass_*​) itself depends on multiple factors, as suggested by Büchi et al..^[5]^ It can be considered a combination of fluid transport in the catalyst layer (*η_Π,CL_*) and the PTL bulk (*η_Π,PTL_*), as well as ionic transport in the catalyst layers (*η_σ,CL_*), as shown in equation (19).

| $\eta_{mass}=\eta_{\sigma,CL}+\eta_{\Pi,CL}+\eta_{\Pi,PTL}$ | (19) |
| --- | --- |

The contribution *η_Π,PTL_* depends, among other factors, strongly on the thickness and porosity of the employed PTL and has been the subject of thorough research.^[3, 5, 14]^ On the other hand, ionic transport is influenced by the amount and distribution of the proton-conducting ionomer.^[15-16]^ An increasing amount of ionomer relative to the catalyst will decrease the overall proton transport resistance, but increase the overall cell potential. It has been claimed that an excessive amount of ionomer will block not only catalytically active sites, increasing the overall kinetic overpotential, but also diffusion paths for incoming water and outgoing gases.

VBA – Combination

The above discussed contributions to the overall cell voltage are summarized in Figure S12. In order to identify the influence of the pore size/surface area of our catalyst materials, some variables have to be eliminated in order to simplify the interpretation of the data (marked with a red frame in Figure S12). The remaining contributions are highlighted with a blue frame. As we employed the same cell configuration for all our experiments losses due to Ohmic resistance of the GDL, PTL and the membrane ($R_{GDL}^{el}, R_{PTL}^{el},R_{memb}$), as well as transport through the PTL and GDL ($\eta_{\Pi,PTL}^{H_{2}O}, \eta_{\Pi,PTL}^{O_{2}},\eta_{\Pi,GDL}^{H_{2}},\eta_{\Pi,PTL}^{H_{2}O}$) similar for all tested materials. The same holds true for the cathode layer and its contributions, including proton and hydrogen transport as well as kinetic overpotential for the HER ($\eta_{HER},\eta_{\sigma,cath}^{H^{+}},\eta_{\Pi,cath}^{H_{2}}, \eta_{\sigma,memb}^{H^{+}}$).

**Supplementary Notes 5: Voltage Breakdown Analysis, Equivalent Circuit Fitting and Distribution of Relaxation Times Analysis (DRT)**

*Kinetic Contributions:* To estimate the influence of the kinetic contribution on the total cell voltage, we determined the Tafel slope of the HFR-free cell potential (Figures S10-S11). The determined Tafel slopes of the materials range between 44 and 51 mV dec^-1^ (Table S3), which falls within the range commonly observed for iridium oxide.^[15, 17]^ Across three measurements of **IrO_x_-IO-(90)**, we determined an average Tafel slope of 45.9 mV dec^-1^ with a standard deviation of 1.2 mV dec^-1^ (Table S2). The kinetic overpotential, calculated from the Tafel slope across the full range of measured geometric current densities, is shown in Figure S13a. Here, **IrO_x_-IO-(120)** exhibits the lowest overpotential at 5 A cm^-^², followed by **IrO_x_-IO-(90)** with values of 337 mV (*b* = 43.9 mV dec^-1^) and 349 mV, averaging 342.1 ± 5.1 mV (*b* = 46.9 mV dec^-1^, average: 45.9 ± 1.2 mV dec^-1^). Both **IrO_x_-IO-(240)** and **IrO_x_-IO-(60)** exhibit kinetic losses of a similar magnitude, ranging between 366 and 370 mV. The highest apparent Tafel slope and, consequently, the highest kinetic overpotential are observed for **IrO_x_-IO-(20)**, with values of 50.8 mV dec^-1^ and 399.5 mV.

The reason for the variation in the Tafel slope and, consequently, the kinetic overpotential is not immediately clear. This variation does not appear to be caused by material properties at the nanometer scale, as no clear correlation is observed between crystallite size (determined from Le Bail fitting, Figure S9) and the Tafel slope (Figure S15). This finding is further supported by the literature.^[18]^ Figure S16 shows a slight negative correlation between the apparent Tafel slope and pore size, while the kinetic contribution reaches a minimum for **IrO_x_-IO-(120)** before increasing again. We hypothesize that these relationships arise from ionomer distribution, which has been shown to strongly influence the kinetics of the oxygen evolution reaction in a PEM electrolyzer.^[15-16, 19-20]^

A larger pore size increases the likelihood of ionomer diffusion into the catalyst particles, resulting in more homogeneous coverage, which subsequently increases the number of available triple-phase boundaries. If, on the other hand, the ionomer cannot penetrate the catalyst pores, it results in an inhomogeneous distribution, with a higher concentration on the outer surface of the particles. This not only blocks active sites and reduces the number of available ionomer-catalyst-electrolyte interfaces but also negatively impacts other factors contributing to the overall cell voltage, which will be discussed in the following chapters. The increased thickness of the ionomer layer on the outside of the catalyst particles is implied by the decreasing anode layer thickness of the mesoporous catalysts compared to the macroporous materials. As the literature suggests, the excessive ionomer will fill up voids between catalyst particles in the catalyst layer, and bind the particles closer together, resulting in a densified electrode.^[15-16]^

*Ohmic Contributions:* The ohmic contribution to the total cell potential is shown in Figure S13a and Table S3. From Figure S17a strong correlation between both ohmic overpotential at maximum current density as well as *R_0_* with both pore diameter as well as surface area can be observed. It has to be noted that the plots of *R_0_* respectively *η_Ohm_* at 5 A cm^-^² vs. the pore size indicate the reaching of a plateau with decreasing pore size. The electrical conductivity of iridium oxide itself should not be a decisive factor, given the comparable crystallite size. Since identical cell hardware, including GDL, PTL, and the cathode layer, as well as similar iridium loadings were used, minor deviations between the prepared MEAs should not account for the observed trend. Larger deviations would have been apparent in the reproducibility measurements discussed in Supplementary Notes 3. Therefore, it is reasonable to rule out both the overall setup and MEA configuration, as well as the catalyst properties as the primary cause of the observed correlation.

Thus, the only possible explanations are an increased contact resistance between the catalyst layer and the porous transport layer, as well as a reduction in conductivity between the catalyst particles that make up the catalyst layer. Due to the previously mentioned lower likelihood of the ionomer penetrating the catalyst particles with decreasing pore size, the resulting polymer film may electrically insulate the particles. This, in turn, increases the contact resistance between the catalyst layer and the porous transport layer, leading to higher ohmic resistance. A similar observation has, among others, made by Bernt et al..^[15]^ When a critical pore diameter is reached, ionomer diffusion becomes more feasible, and the polymer no longer impedes inter-particle conductivity or increases the contact resistance between the PTL and the catalyst layer, as indicated by the behavior of **IrO_x_-IO-(120)** and **IrO_x_-IO-(240)** shown in Figure S17.

*Catalyst layer Resistance:* The contribution of the catalyst layer resistance to the overall cell voltage is shown in Figure S13a. The three mostly macroporous materials (**IrO_x_-IO-(240)**, **IrO_x_-IO-(120)**, and **IrO_x_-IO-(90)**) show similar values of around 49 mV, which lie within the margin of error (3.1 mV). The two more mesoporous materials (**IrO_x_-IO-(60)** and **IrO_x_-IO-(20)**), on the other hand, have comparable values of about 55 mV at 5 A cm^-^². From the correlation plots of both *R_CL_* and *η_CL_* with the pore size and surface area, no clear trend can be observed, except for the previously mentioned apparent dependence on pore type (mesoporous vs. macroporous; Figure S18).

*Mass Transport Resistance:* At last, the mass transport overpotential is shown in Figure S13a. Here material **IrO_x_-IO-(240)** shows the lowest overpotential of < 5 mV, followed by **IrO_x_-IO-(120)** (6 mV) and **IrO_x_-IO-(90)** (12 mV ± 5.4 mV) and **IrO_x_-IO-(60)** (7 mV). A strong increase is visible for **IrO_x_-IO-(20)** reaching values of around 56 mV. The correlation plot (Figure S19) of *η_mass_* vs. pore size can be divided into two parts: A slight non-negative correlation of the mainly macroporous materials, with average pore diameter between 60-240 nm can be observed. With further decrease in pore size, as visible for **IrO_x_-IO-(20)** the mass transport overpotential strongly increases. Here, two factors may be decisive. First, the diffusion of the ionomer in particular into the particles may be more easily facilitated for larger pores, as already indicated from the discussion of the ohmic contribution. Oxygen bubbles, on the other hand, may become trapped inside the catalyst with a small pore diameter. Furthermore, due to the inverse proportionality of the capillary pressure $P_{C}$ to the pore diameter (*d_c_*) (20), the gas pressure inside the pore has to be higher for mesopores than for macropores. This in turn will result in a higher mass transport resistance.^[16]^

| $P_{C}\propto\frac{1}{d_{c}}$ | (20) |
| --- | --- |

Because the tortuosity of the inverse opals is theoretically independent of pore size, the observed trend should not be attributed to the diffusion of water or dissolved oxygen. Furthermore, the previously proposed ionomer film on the exterior of the catalyst particles may hinder the diffusion of both oxygen and water. In contrast, no clear correlation between surface area and mass transport resistance can be identified (Figure S19).

*Equivalent Circuit Modelling:* The impedance spectra recorded at 1.5 V are shown in Figure S13b. For all materials except **IrO_x_-IO-(20)** and **IrO_x_-IO-(60)**, two distinct semicircles can be observed, as shown in the inset of Figure S13b.^[21]^ The high-frequency arc, which is commonly assigned to cathode processes, is only slightly visible or absent for **IrO_x_-IO-(20)** and **IrO_x_-IO-(60)**. The anodic charge transfer resistance *R_CT_*, as well as the corresponding double-layer capacitance C_dl_ - typically assigned to the low-frequency semicircle - were analyzed by fitting an equivalent circuit to the experimental data (Figure S10e). The anodic charge transfer resistance reaches a minimum of approximately 116 mΩcm² for **IrO_x_-IO-(120)**, closely followed by **IrO_x_-IO-(90)** (124 mΩcm²). Both **IrO_x_-IO-(240)** and **IrO_x_-IO-(60)** show higher values of 147 mΩcm² and 178.2 mΩcm², respectively. As expected from the previous analysis, **IrO_x_-IO-(20)** shows the highest anodic *R_CT_* of 333 mΩcm². Within the margin of error, a comparable trend is observed in the anodic double-layer capacitances C_dl_ (Figure S13b).

A notable difference is observed between the mostly macroporous materials (**IrO_x_-IO-(240)**, **IrO_x_-IO-(120)**, and **IrO_x_-IO-(90)**) and the mesoporous materials (**IrO_x_-IO-(60**) and **IrO_x_-IO-(20)**) (Table S3). The correlation plots of double-layer capacitance and anode charge transfer resistance against the pore size respectively, surface area confirm this trend (Figure S20).

*Distribution of Relaxation Times Analysis (DRT):* The DRT analysis was performed using the pyDRTtools package, applying Tikhonov regularization. Gaussian functions were chosen for the discretization method, and both the real and imaginary components of the impedance data were used. Inductance was included in the analysis. Following recent literature, the regularization parameter was set to 0.0001.^[22]^ For the analysis, impedance spectra measured at current densities of 1, 2, 3, 4, and 5 A cm^-^² were selected (Figure S23). To ensure the validity of the impedance data for each measurement, the Kramers–Kronig test was performed as described in the previous section (Figure S24). The resulting DRT data for the five current densities of each material are shown in Figure S25.

Depending on the specific sample and applied current, between three and five peaks can be observed. According to recent studies on the application of DRT in PEM electrolysis, these peaks, depending on their time constant or frequency, can be associated with specific electrochemical processes. In general, 4–5 peaks are observed, corresponding, with decreasing frequency (increasing time constant), to ionic transport processes (proton transfer), charge transfer and kinetic processes (HER/OER), and mass transport processes related to the electrolyte and gas bubbles. The exact assignment of peaks and their respective frequency ranges is not yet fully established, due to the relative novelty of DRT in PEM electrolysis. Nevertheless, proton transfer processes typically occur around 2000–5000 Hz, electrode processes (HER/OER) around 100–1000 Hz, and mass transport-related processes around 0.1–60 Hz.^[2-22]^ These values may vary between different literature studies.

A more detailed analysis at high current densities shows that the peak intensity associated with proton transport (~10000 Hz) decreases slightly with increasing current density, while its characteristic frequency remains largely unchanged (Peak 1, Figure S25).^[23]^ Charge transfer processes related to HER and OER are observed around 2000 Hz and exhibit a strong decrease in intensity as well as a shift toward higher frequencies (Peak 2, Figure S25). This shift leads to the merging with Peak 1.

Mass transport-related signals (Peak 5) with low intensities are present at all current densities at frequencies of around 1 Hz. The literature indicates that this peak corresponds to gas transport at the PTL/flow field interface.^[23]^ Since neither the PTL nor the flow field were varied in our experiments, a strong increase in peak intensity is not expected. However, Alia et al. assign signals at this relaxation time to mass transport processes within the catalyst layer.^[24]^ While the exact origin and intensity increase of this peak cannot yet be fully explained, its observation clearly indicates a negative effect of the pore size of **IrO_x_-IO-(20)** on overall mass transport properties.

In addition to the peaks discussed above, two further signals appear at higher current densities, most pronounced for **IrO_x_-IO-(60)** and **IrO_x_-IO-(20)**. Literature suggests that Peaks 3 and 4 arise from gas transport either within the catalyst layer or at the catalyst layer/PTL interface, although their exact origin remains unresolved. As in the case of Peak 5 for **IrO_x_-IO-(20)**, these observations highlight the influence of pore structure on mass transport behavior.

The direct comparison of the DRT analysis at maximum current density clearly demonstrates, that with decreasing pore size the contribution of mass transport severely increases.


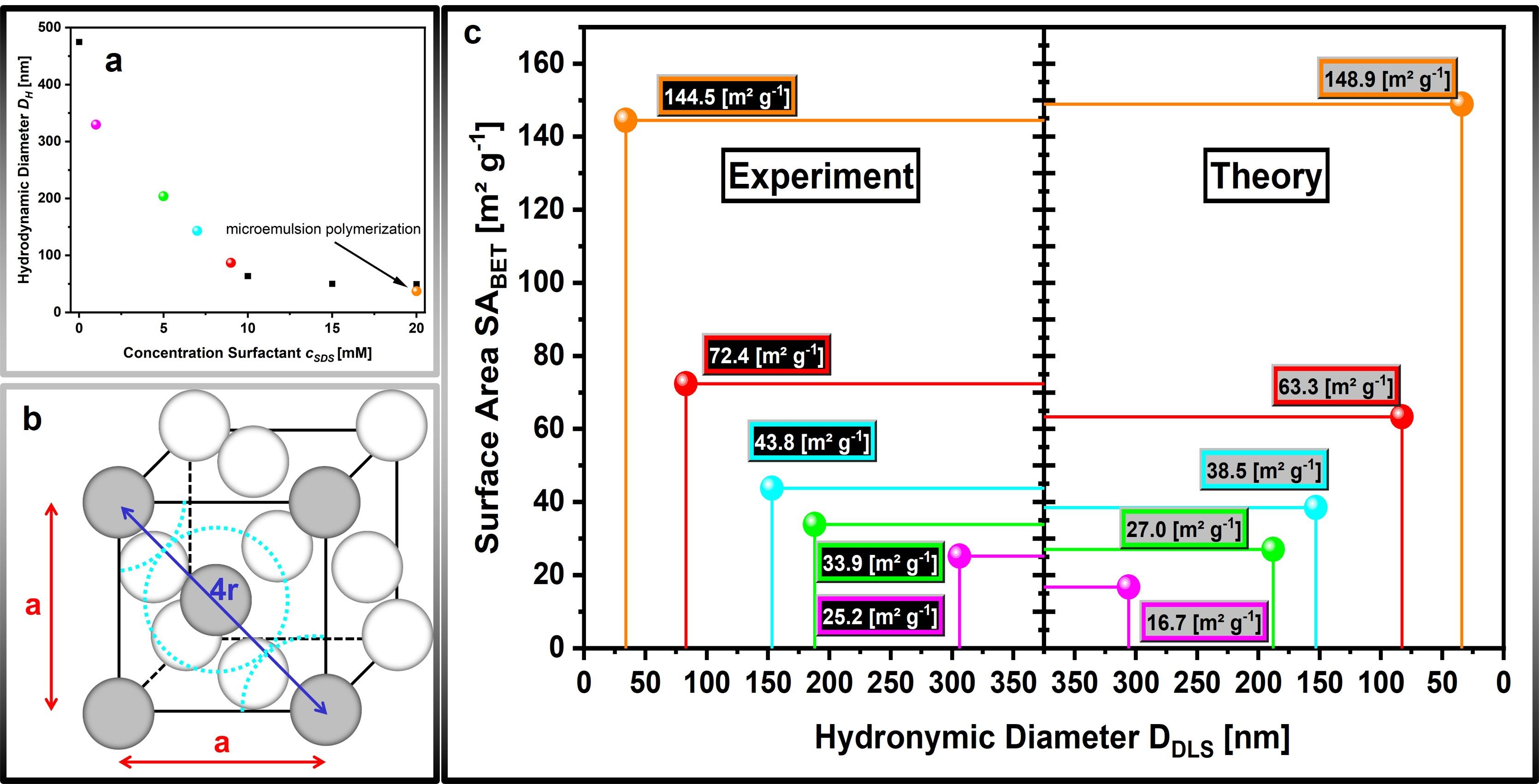


**Figure S1: Influence of the surfactant concentration on the polystyrene size and its surface area.** a: Dependence on the hydrodynamic diameter determined by dynamic light scattering of the synthesized polystyrene microspeheres with respect to the surfactant concentration. b: Schematic representation of the face centered cubic package*.* c: Direct comparison of measured and calculated surface area of the pristine polystyrene colloidal crystals.


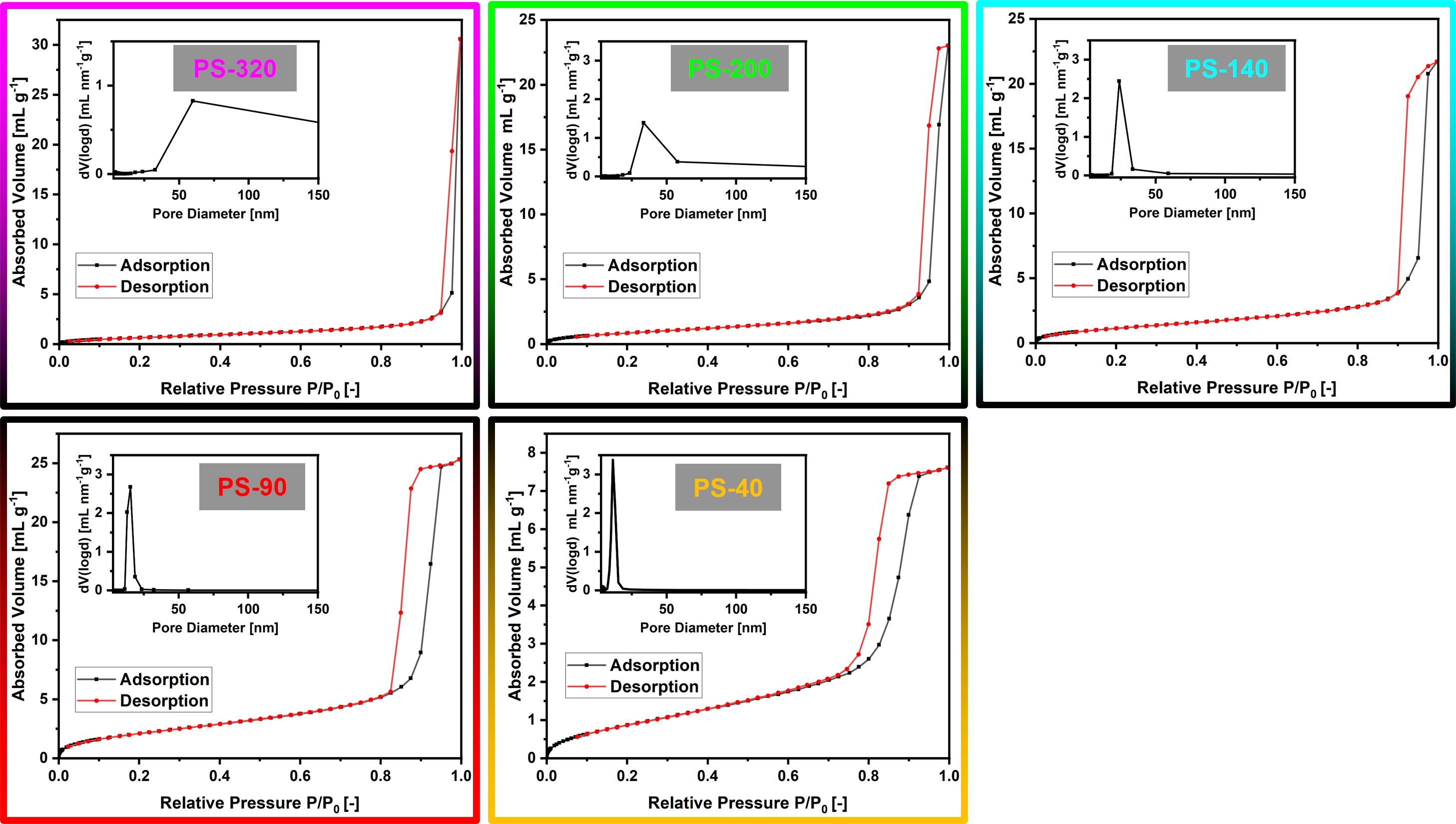


**Figure S2:** N_2_-Physisorption isotherms of the pristine polystyrene colloidal crystals **PS-CC**. Inserts show the BJH-pore size distribution calculated from the desorption branches.


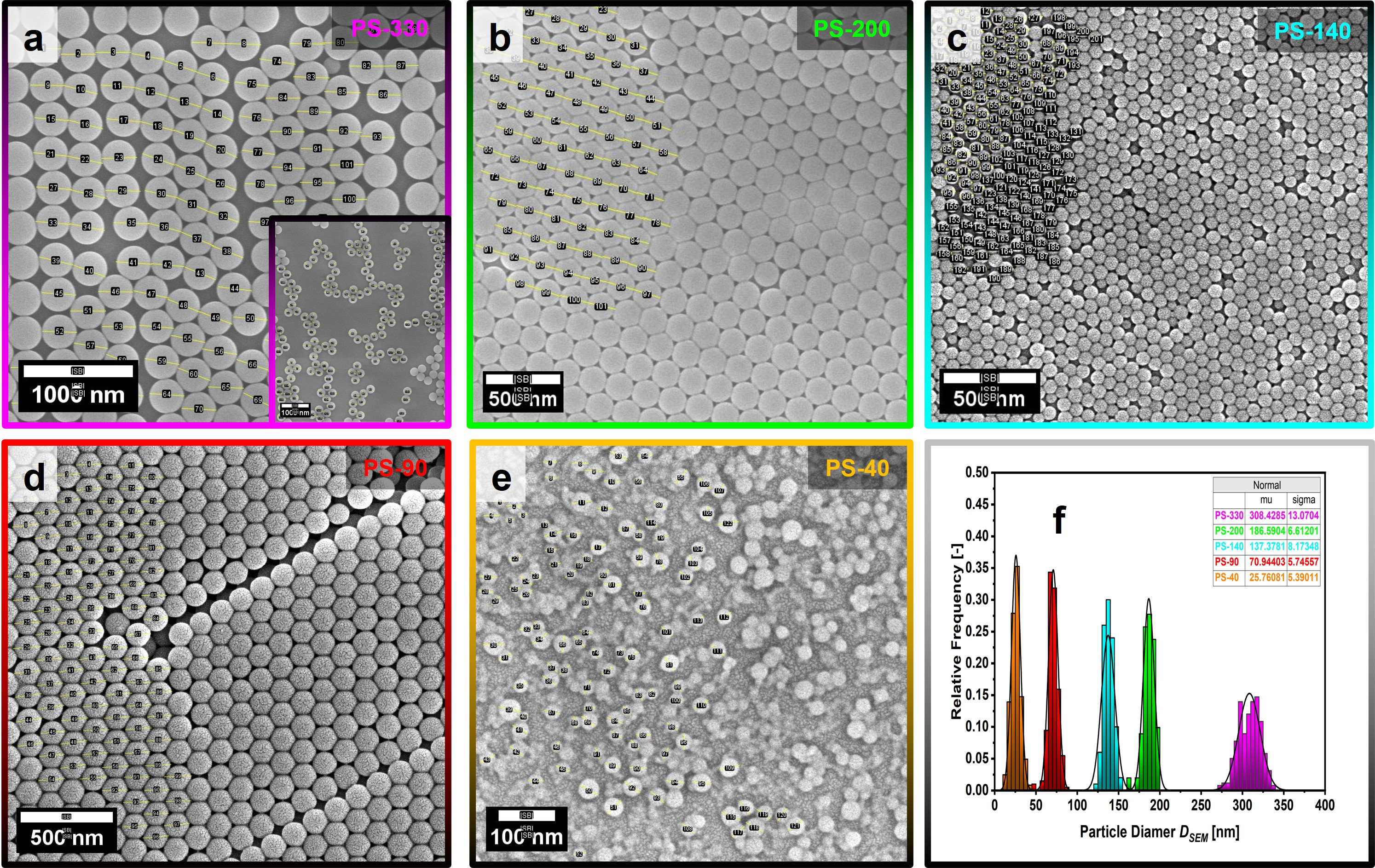


**Figure S3:** a-e: Scanning electron micrographs of the pristine polystyrene colloidal crystals. f: Particle size distribution (normal distribution) of a sample size of at least 100 spheres.


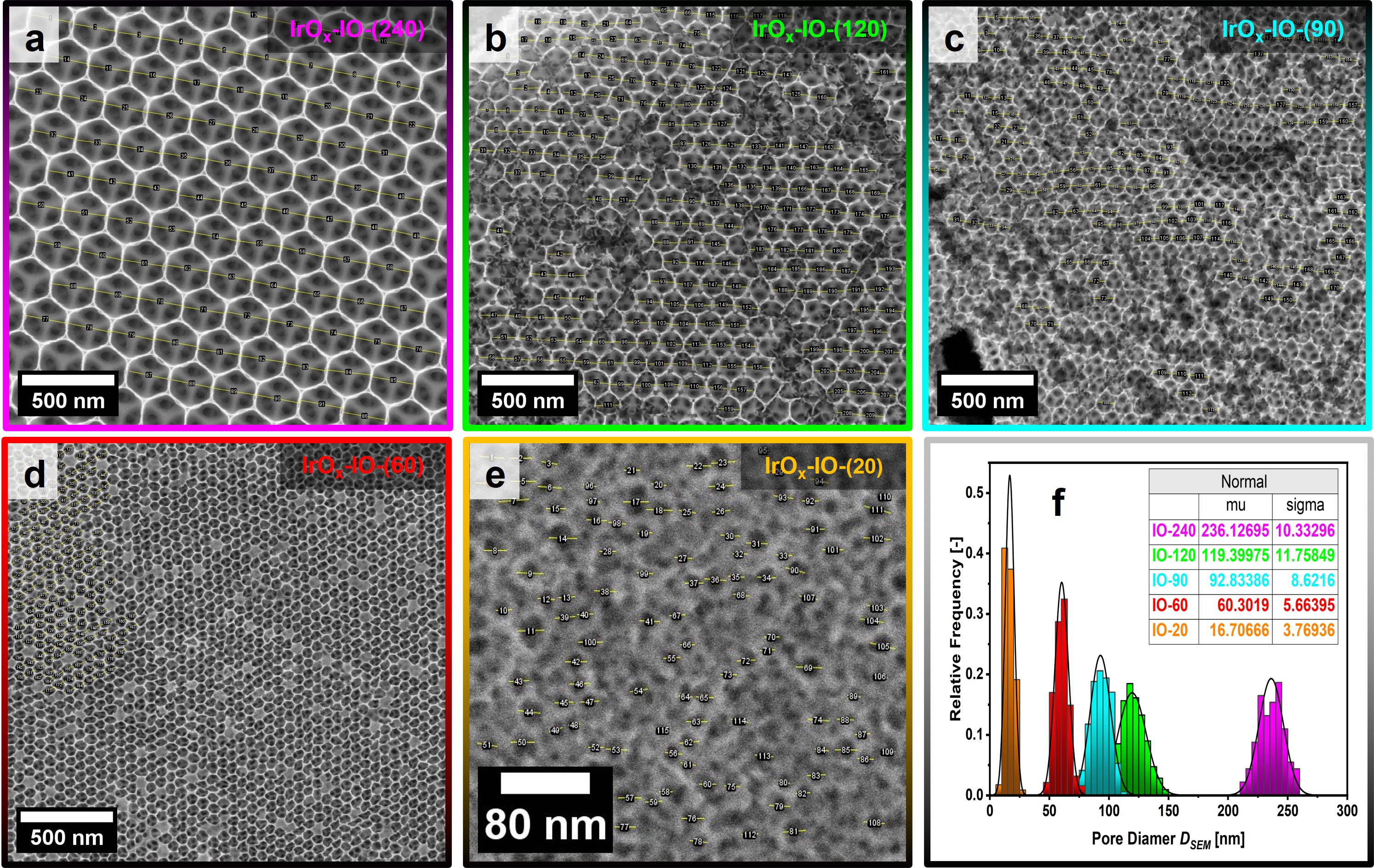


**Figure S4:** a-e: Scanning electron micrographs of porous iridium oxide catalysts. f: Pore size distribution (normal distribution) of a sample size of at least 100 pores.


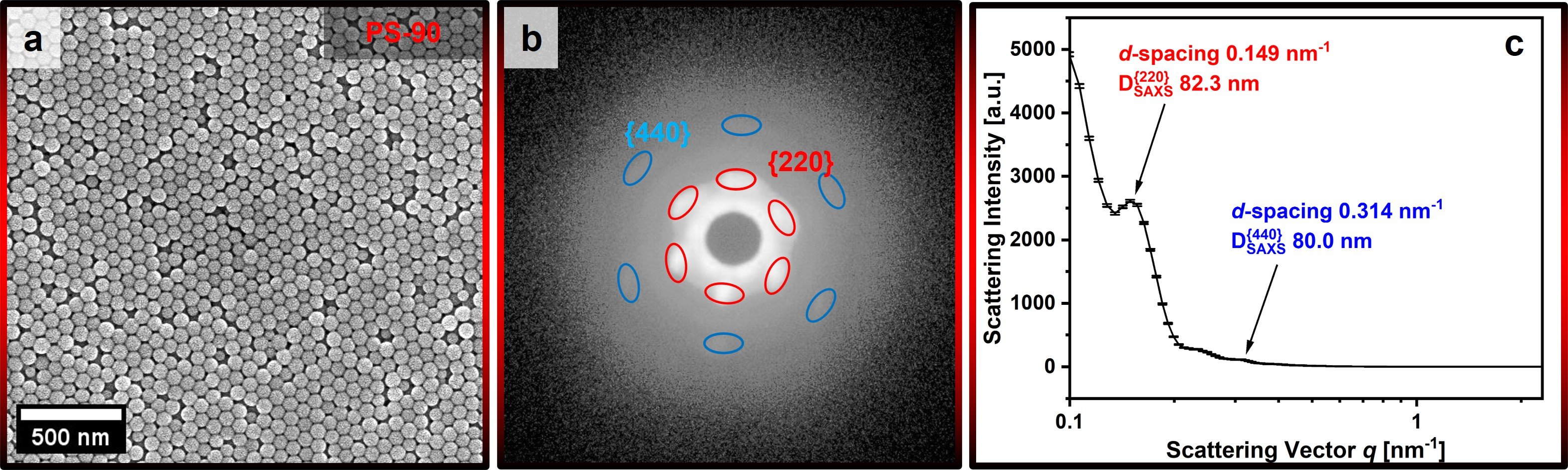


**Figure S5:** a: Scanning electron micrographs of **PS-90**. b: Small angle scattering pattern of **PS-90** recorded using a laboratory device (Bruker Nanostar). c: Integrated, semilogarithmic representation of the scattering pattern (b). The respective set of planes {440} and {220} are annotated.


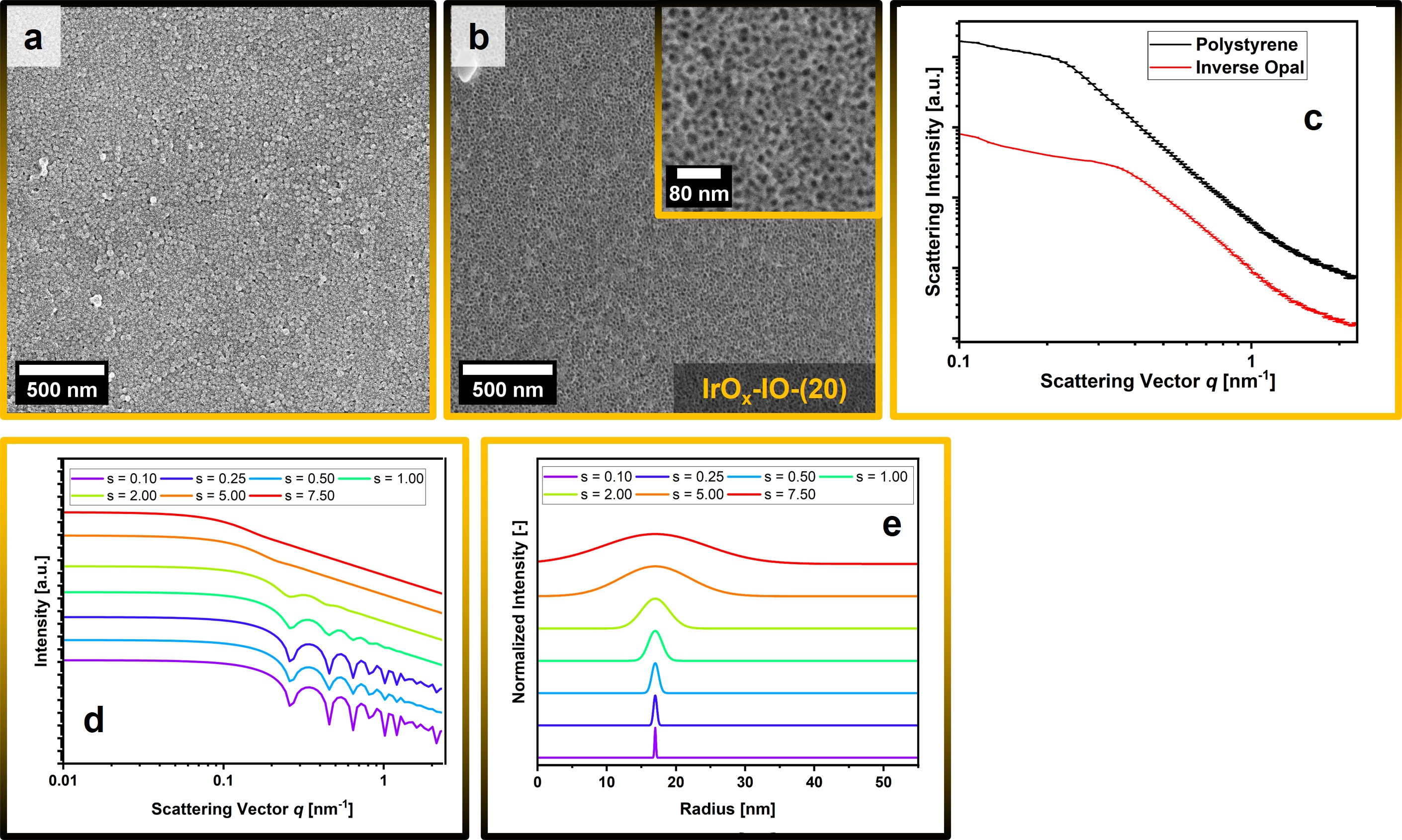


**Figure 6:** a-b: Scanning electron micrographs of **PS-40** and **IrO_x_-IO-(20)**. c: Integrated, double logarithmic scattering curves of the both template and porous material shown in a-b. d-e: Simulated scattering curves for spherical particles (r = 17 nm) and their corresponding size distributions**.** A normal distribution with no structure was assumed. With increasing broadness the features in the scattering curves disappear, justifying the featureless curves shown in a.


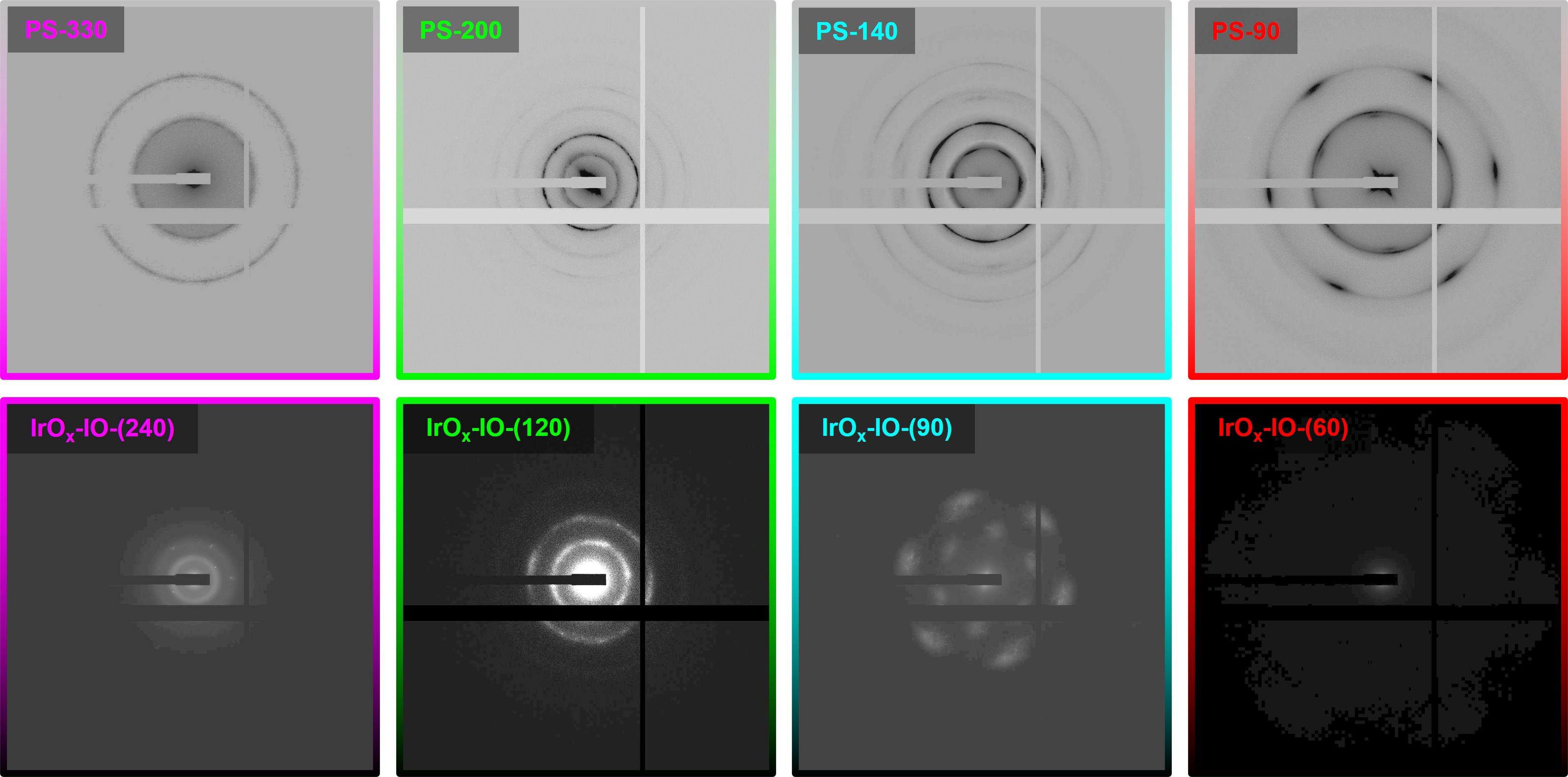


**Figure S7:** a-b: Small angle scattering patterns of polystyrene (top row) and inverse opals (bottom row) recorded at a synchrotron facility (ESRF, ID02).


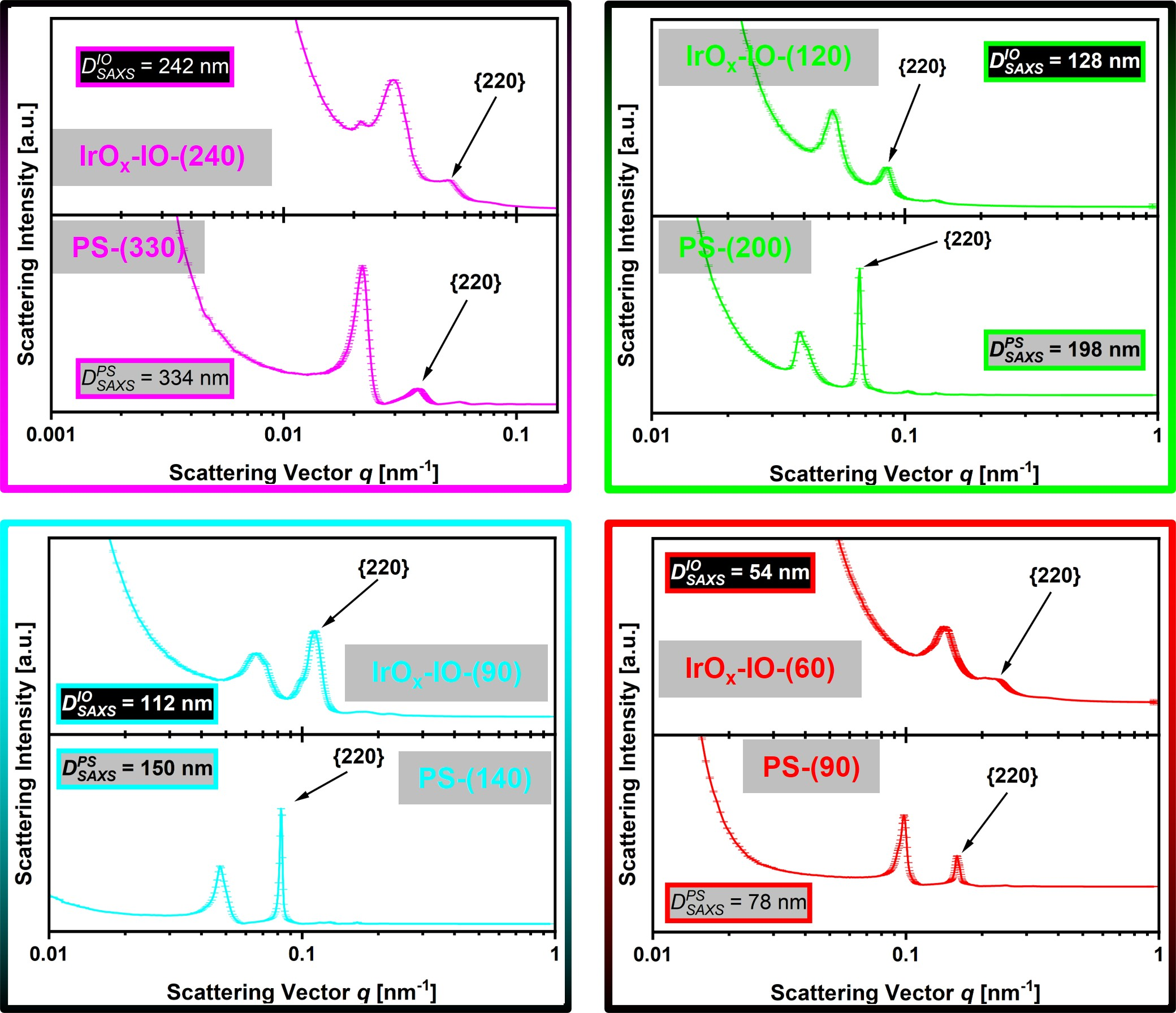


**Figure S8:** Integrated, semilogarithmic representation of the scattering pattern (Figure S7). The respective set of planes {440} and {220} are annotated.
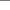


**Table S1:** Summarized results of the particle/pore size distribution.


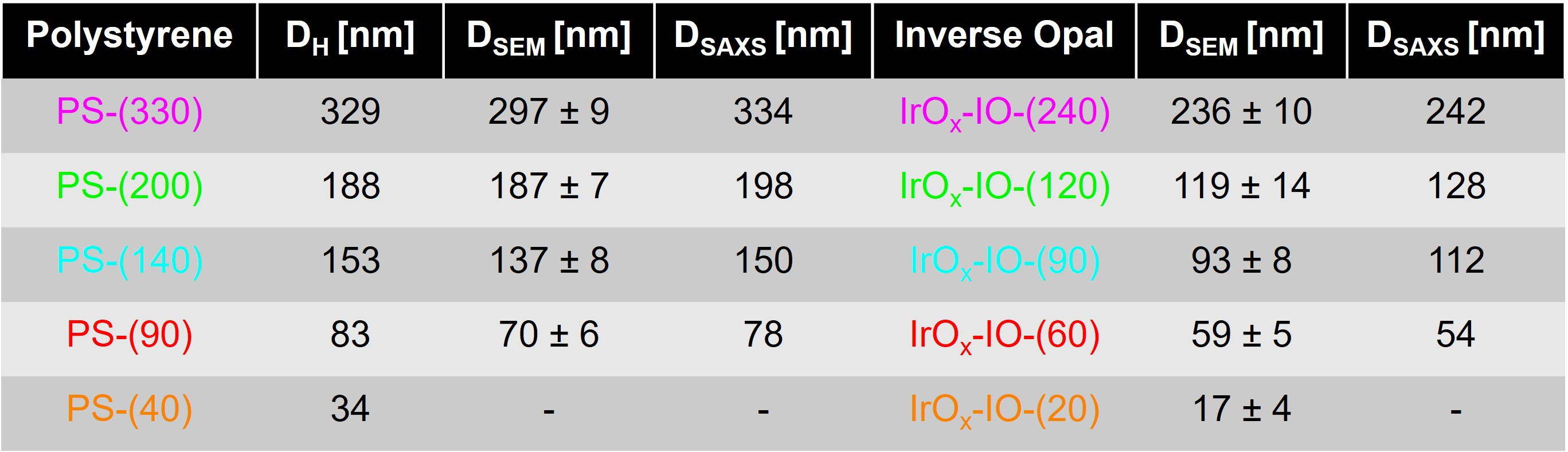


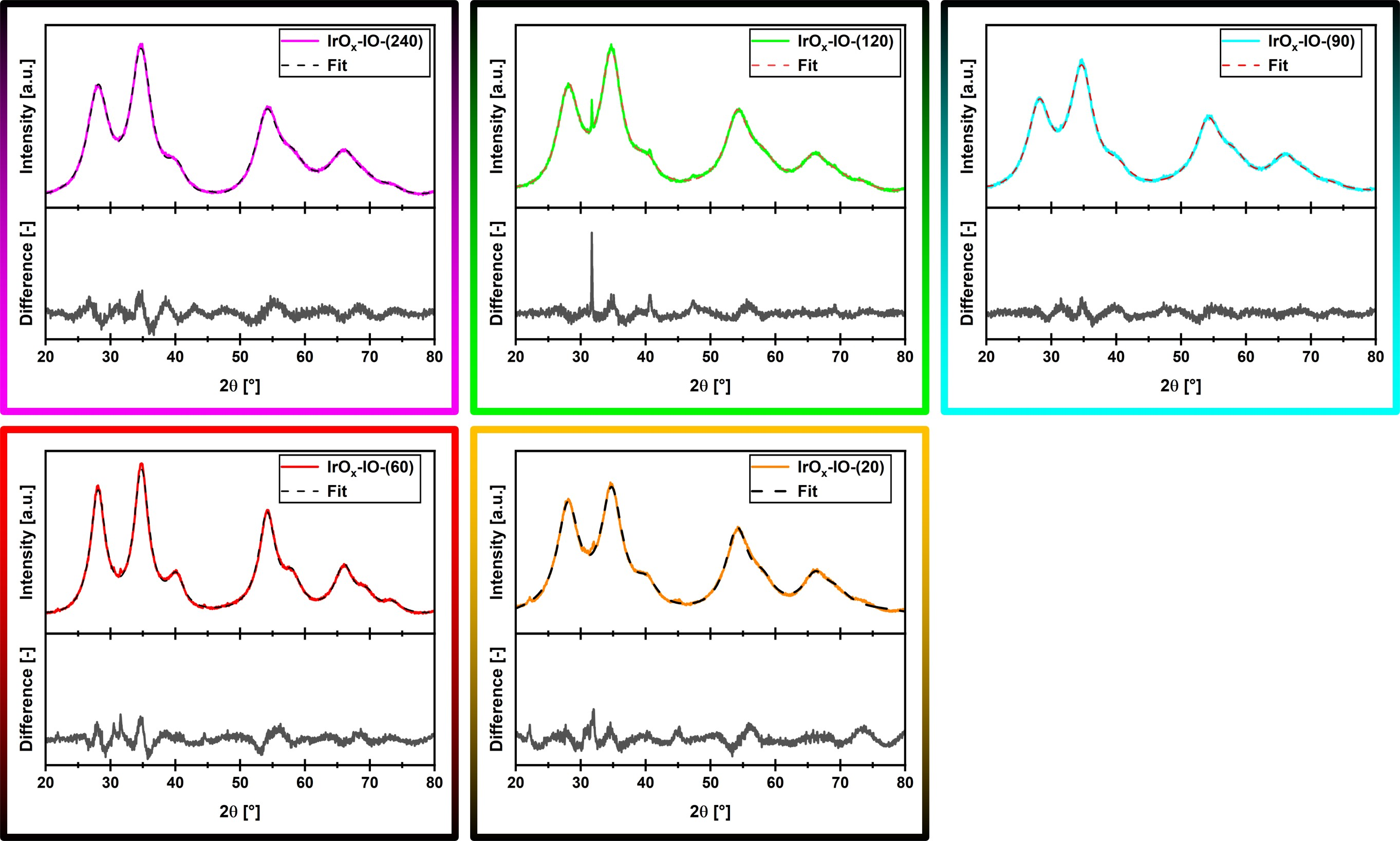


**Figure S9:** Le Bail fittings of the powder X-Ray diffractograms of the five porous iridium oxide materials


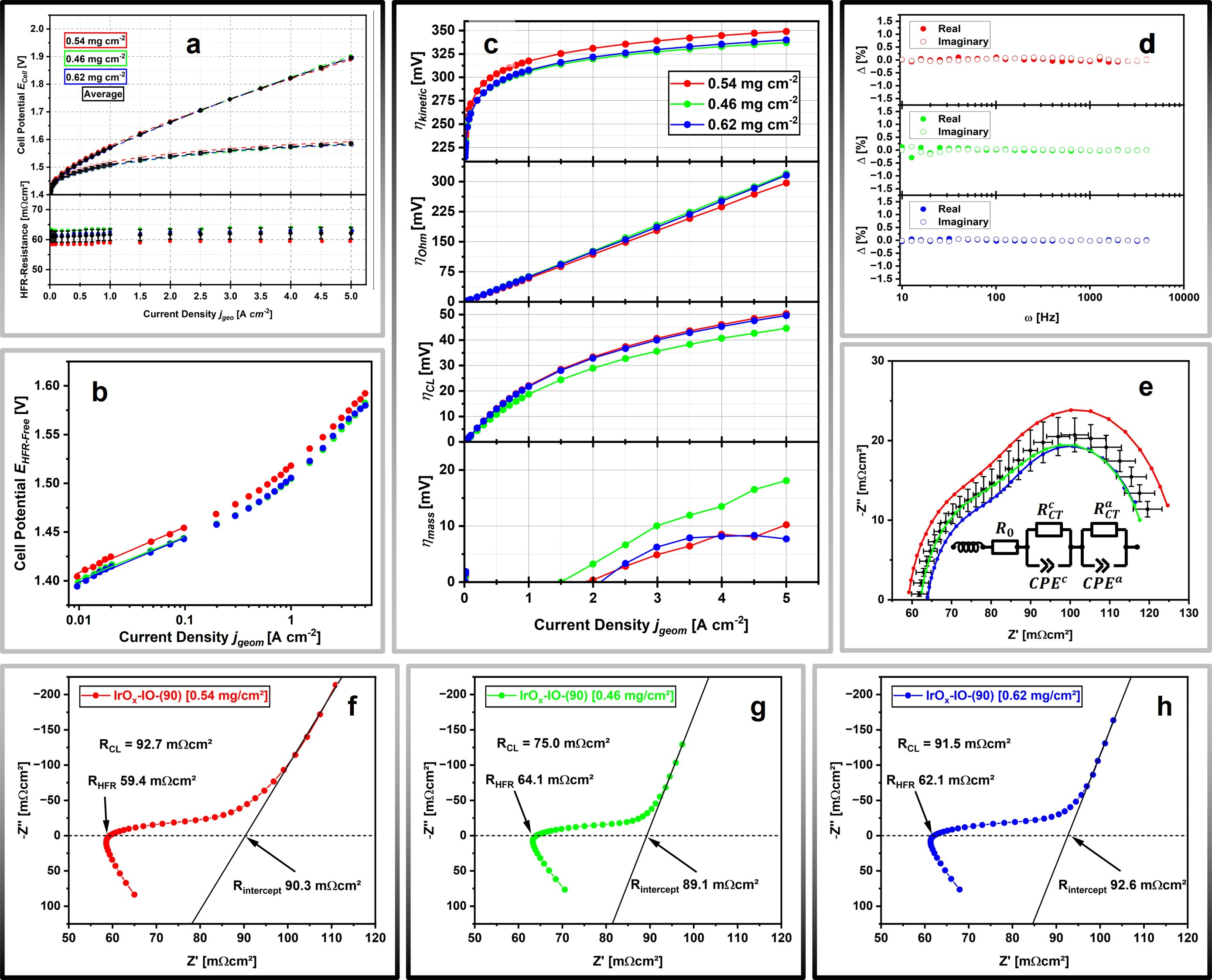


**Figure S10: Reproducibility study of IrO_x_-IO-(90) of three sligthly varying loadings.** a: Polarization curves including the respective HF-resistances and their averages. b: Tafel plot. c: Voltage Break Down analysis. d: Kramer Kronig test of the impedance spectra shown in e. e: Potentiostatic impedance spectra @ 1.5 V and the equivalent circuit fit of the three samples including their average. The EC is shown included. f-h: Impedance spectra at 1.25 V of the three samples including the fit to determine the catalyst layer resistance *R_CL_*.

**Table S2:** Results for the reproducibility study of **IrO_x_-IO-(90)**


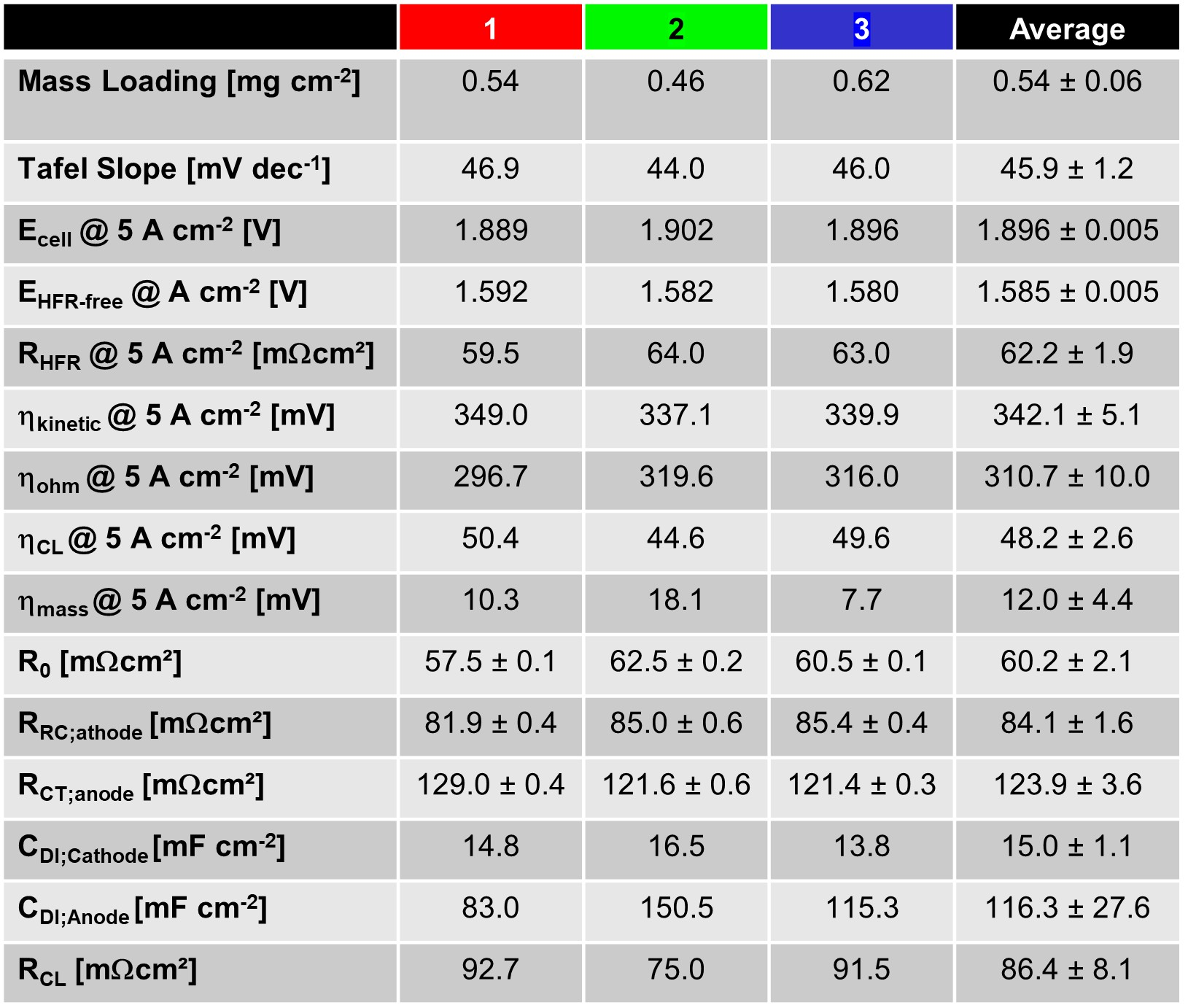


**Table S3:** Physical and electrochemical properties of the investigated inverse opals.


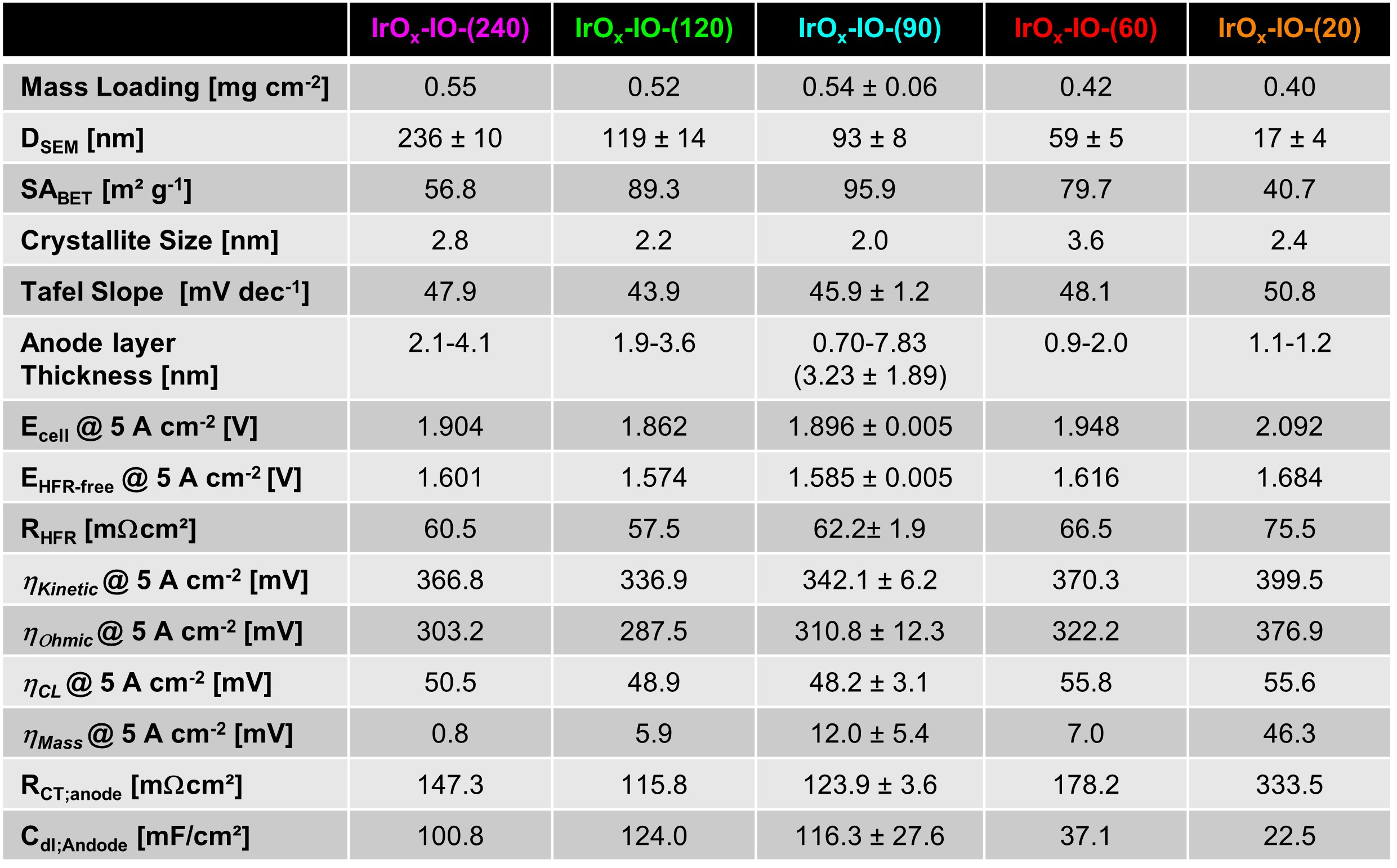


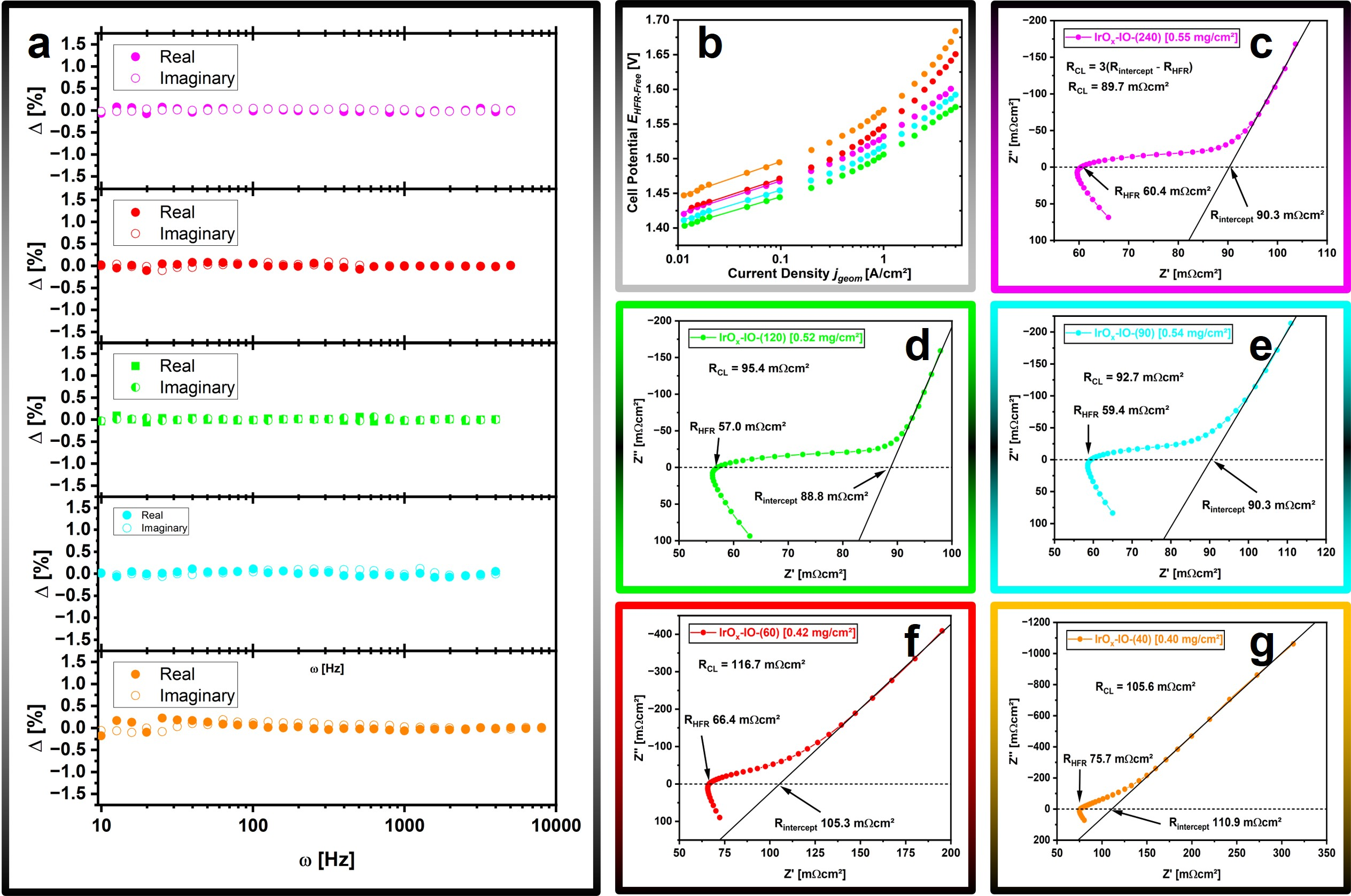
 **Figure S11:** a: Kramer Kronig test for the five different materials of different pore size b: Tafel plots. c-g: Impedance spectra @ 1.25 V of the three samples including the fit to determine the catalyst layer resistance *R_anode;CL_*.


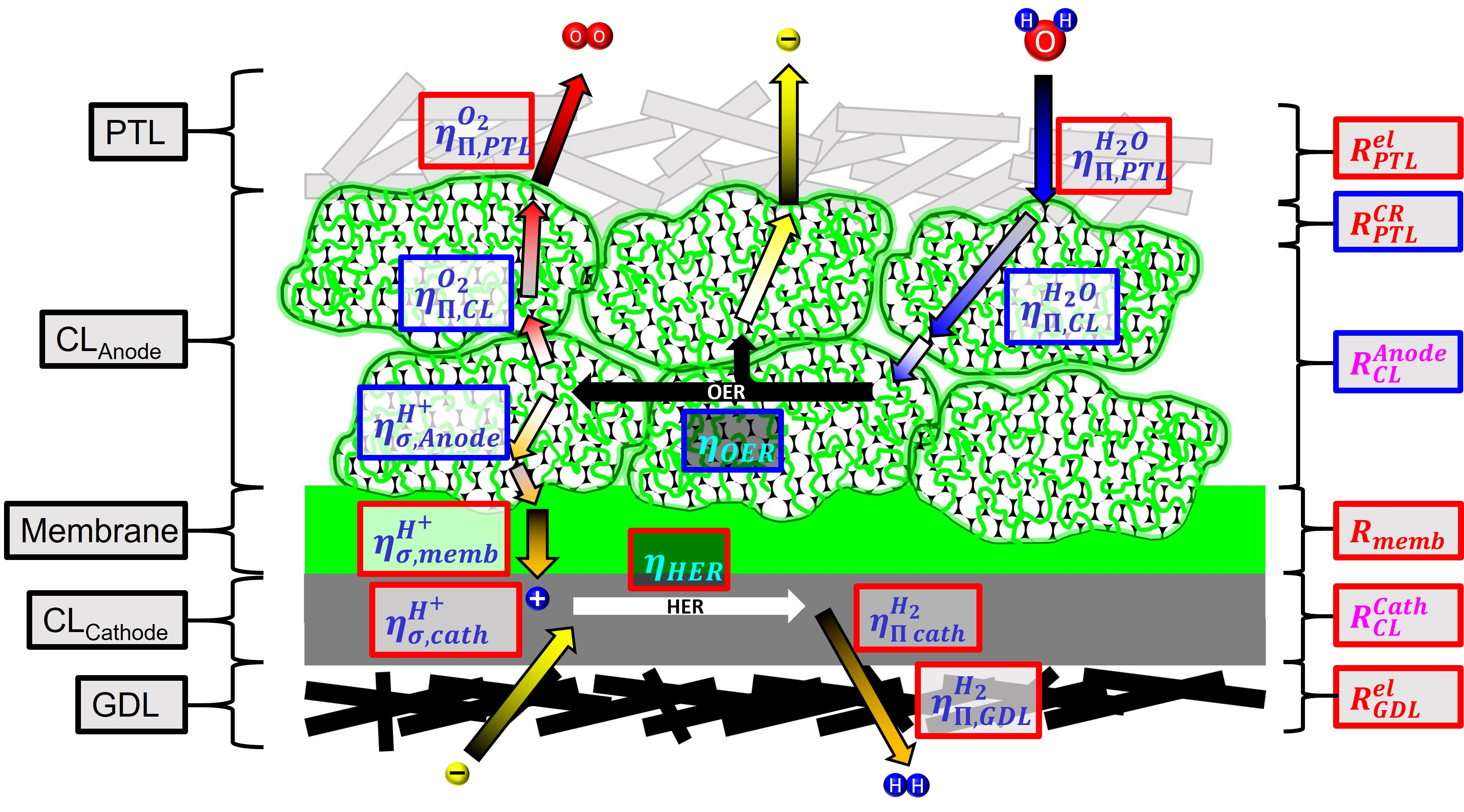


**Figure S12:** A typical membrane electrode assembly consists of the porous transport layer (PTL), the anodic/cathodic catalyst layers (CL_anode/cathode_), the proton exchange membrane and the gas diffusion layer (GLD). Ohmic and kinetic contributions to the overpotential are marked in red respectively in teal. The catalyst layer resistance is marked in magenta. The for the analysis relevant contributions are shown in blue. Neglectable contributions for the comparative evaluation are highlighted with red boxes.


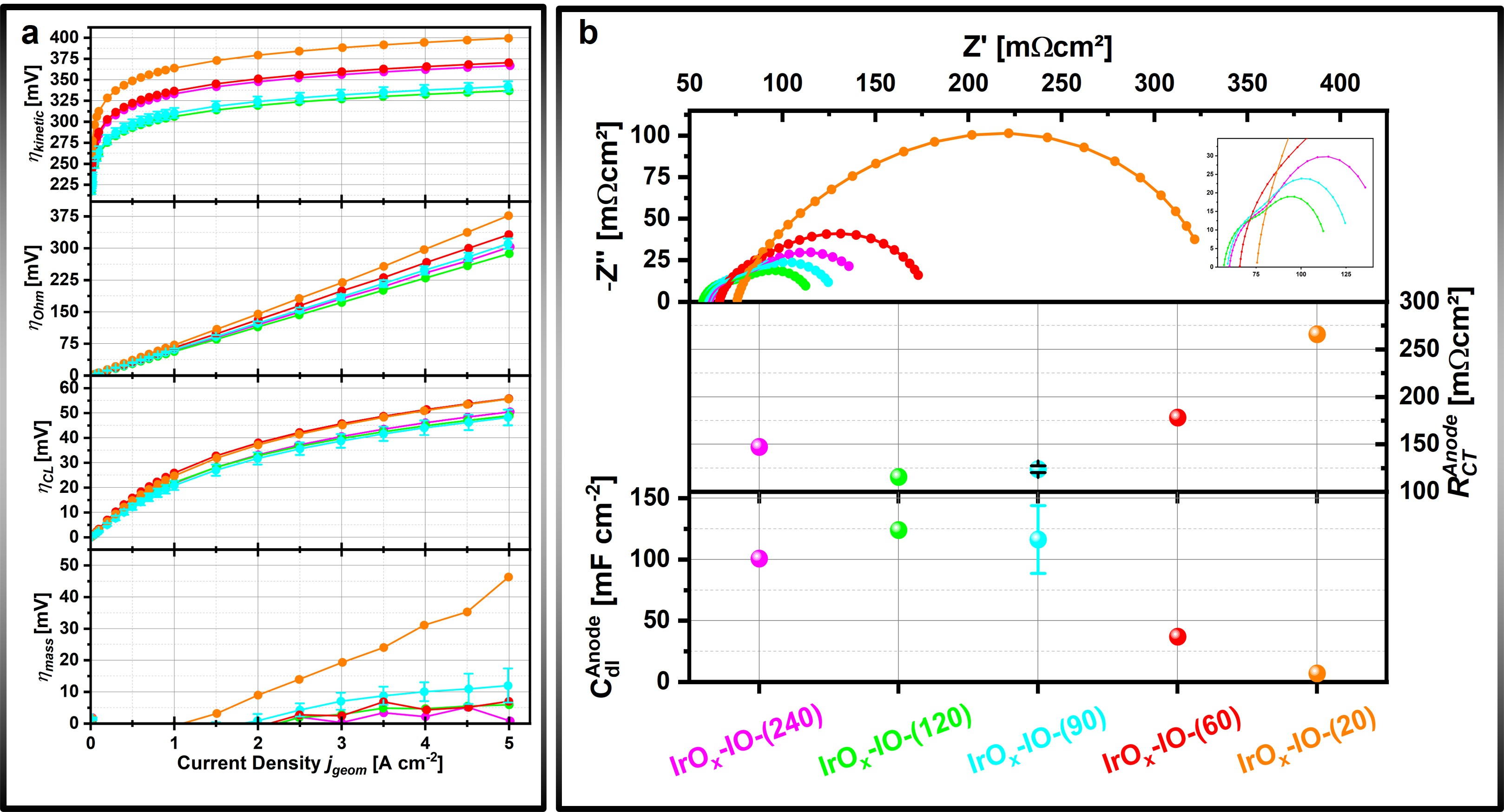


**Supplementary Figure 13:** a: Voltage breakdown analysis of the in a shown polarization curves. Top: kinetic overpotential. Top-Middle: Ohmic overpotential. Bottom-Middle: catalyst layer overpotential. Bottom: mass transport overpotential. b: Results of the potentiostatic impedance spectroscopy @ 1.5 V. Top**:** Nyquist plot of the five investigated materials. Middle: Anodic charge transfer resistances by equivalent circuit fitting. Bottom: Anode double layer capacitances determined from the equivalent circuit fit.


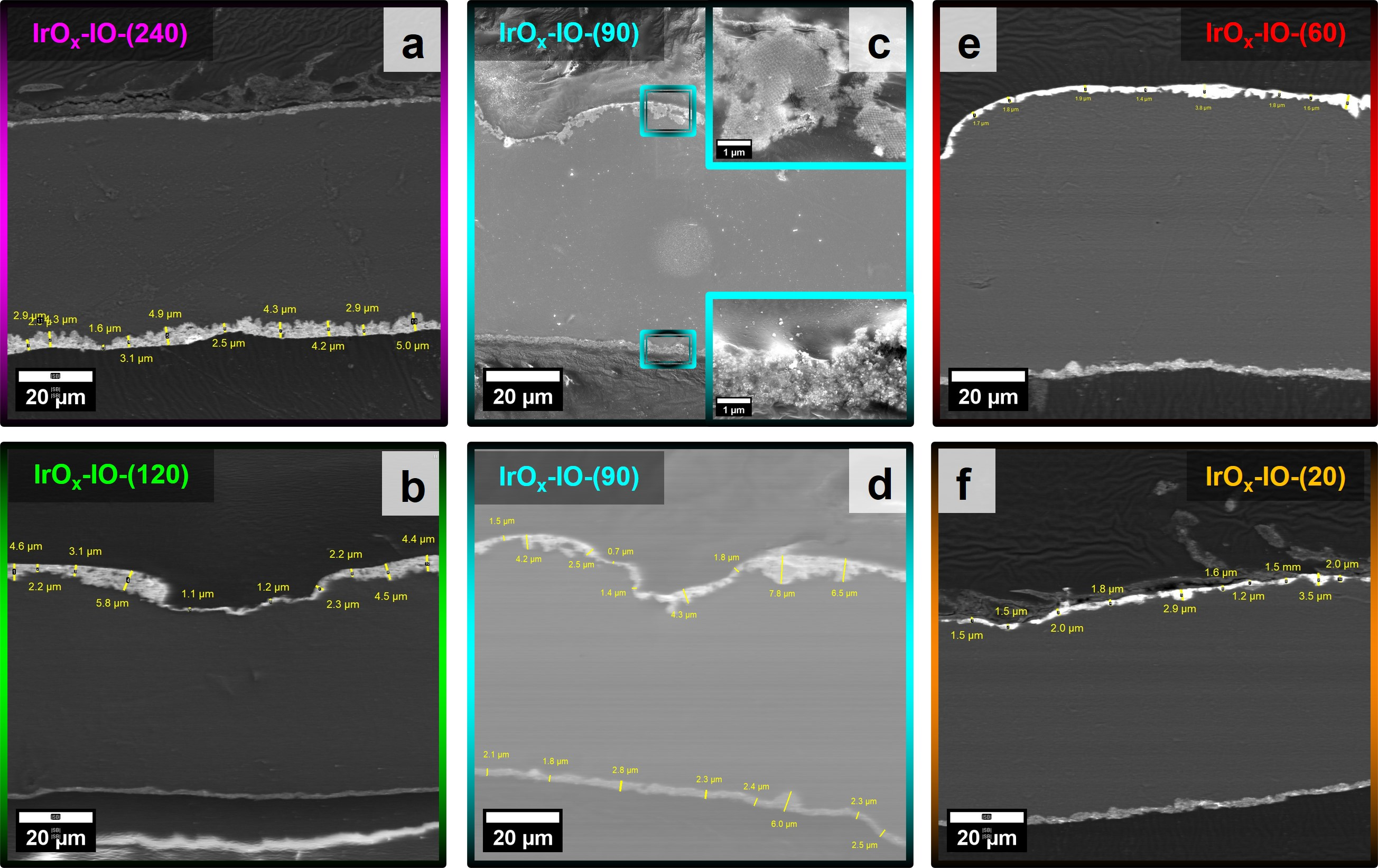


**Figure S14:** Cross section micrographs of the five different **IO** materials recorded using: a-b, d-f: back scattering electron detector; c: inlens detector. Inserts: Zoom of both anode and cathode catalyst layer.


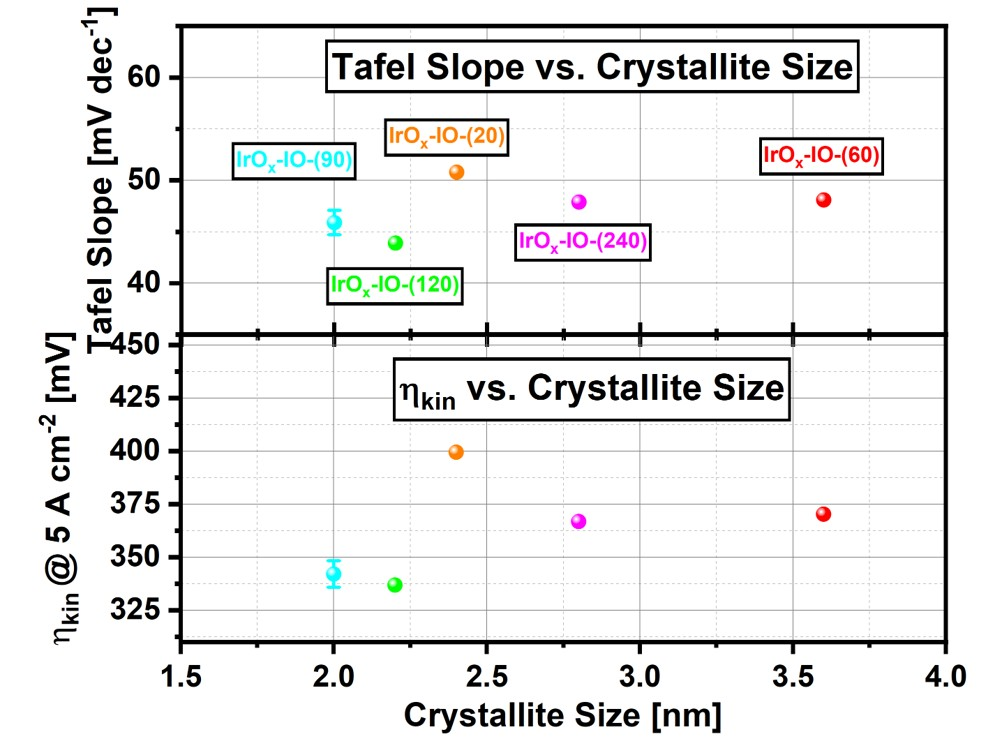


**Supplementary Figure 15:** Correlation plots of Tafel slope and kinetic overpotential @ 5 A cm^-^² vs. crystallite size.


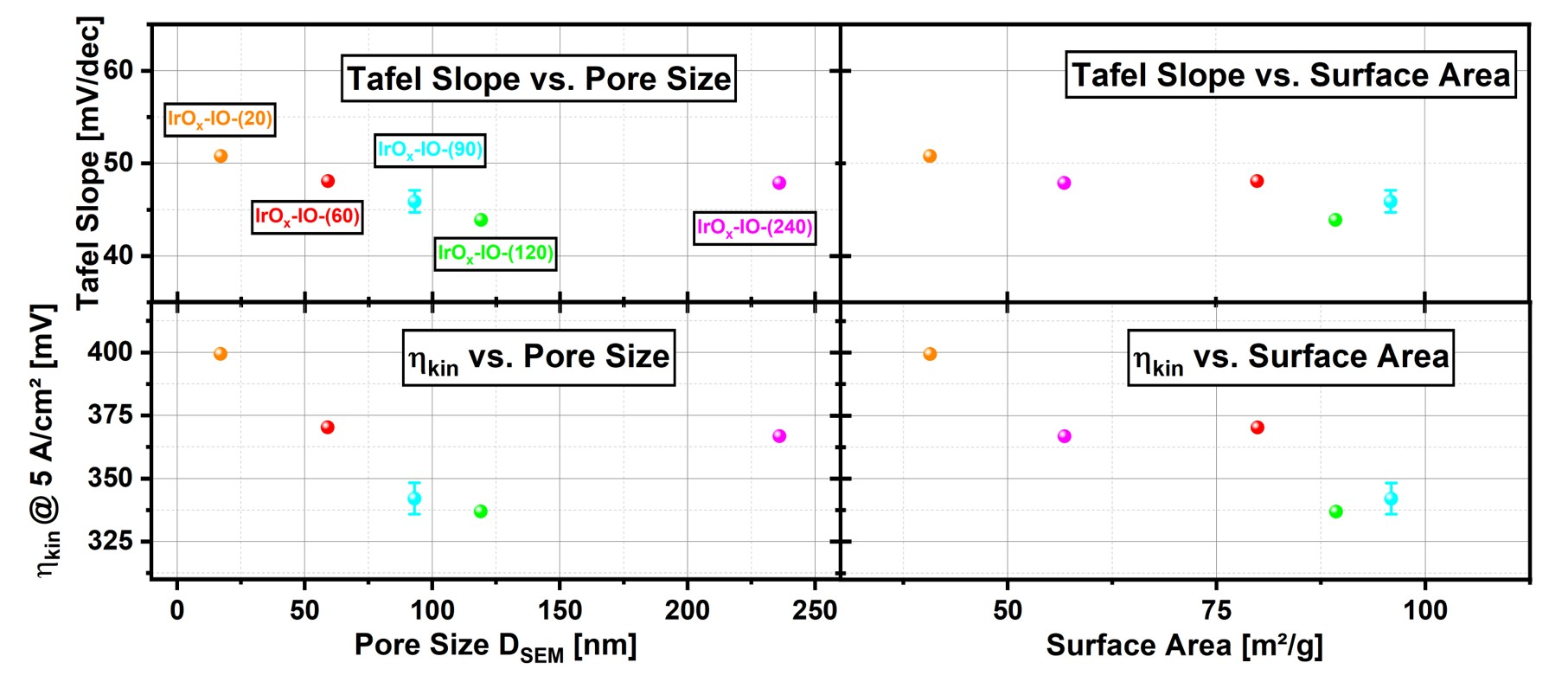


**Figure S16:** Correlation plots of Tafel slope and kinetic overpotential *η_kinetic_* @ 5 A cm^-^² vs. pore size and surface area.


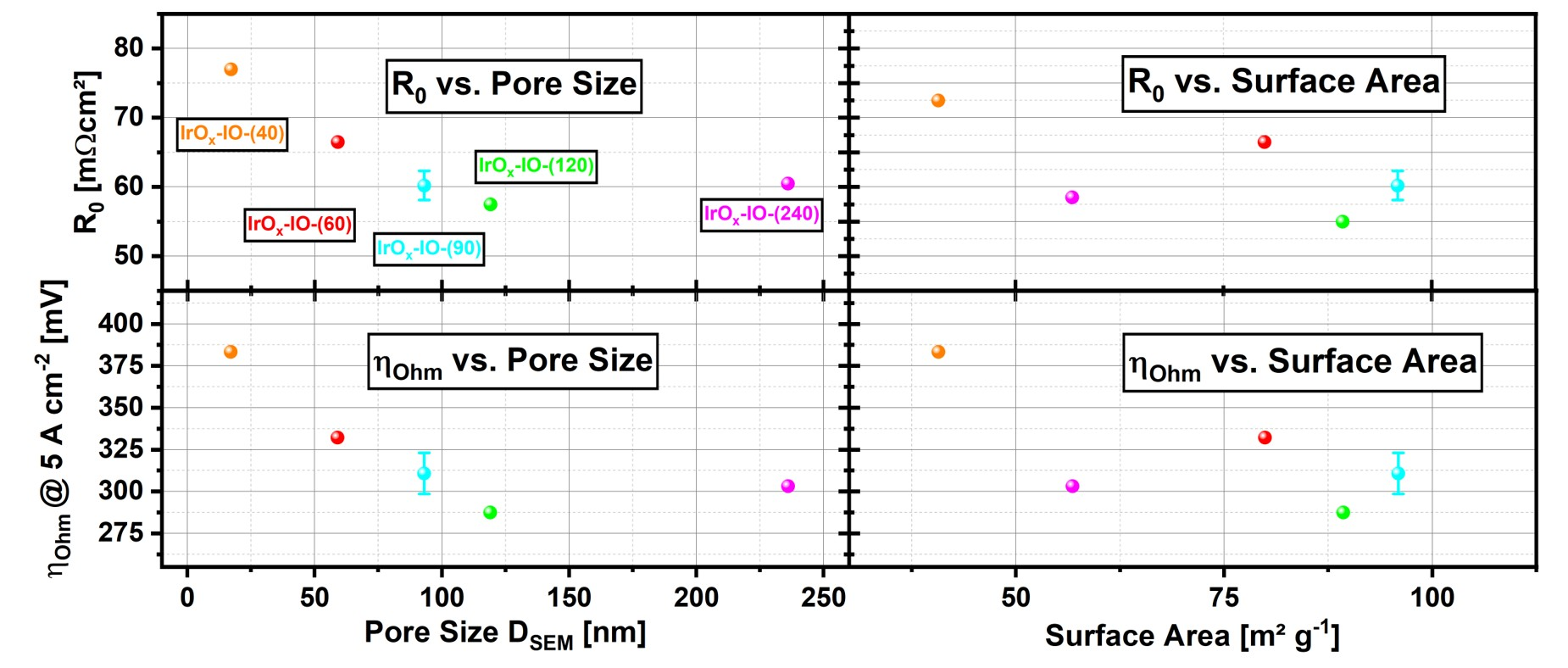


**Figure S17:** Correlation plots of high frequency resistance *R_0_* and ohmic overpotential *η_Ohm_* @ 5 A cm^-^² vs. pore size and surface area.


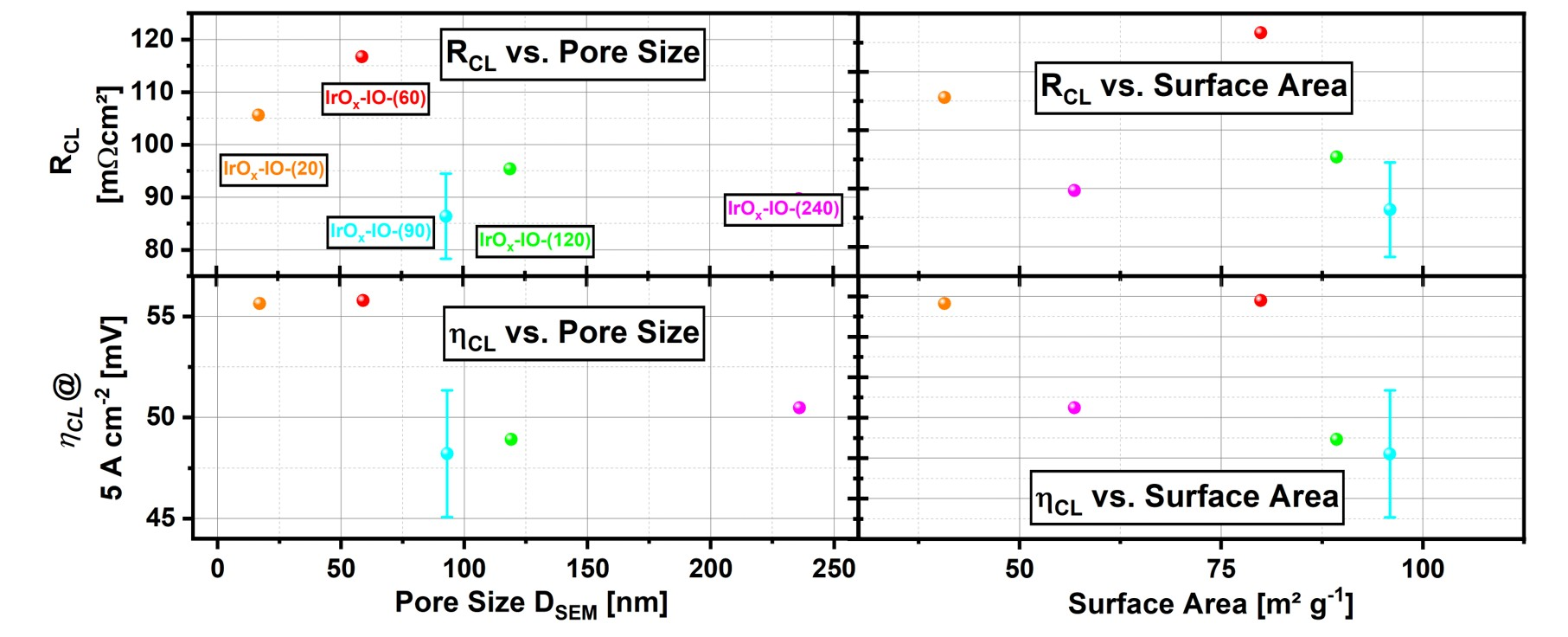


**Figure S18:** Correlation plots of catalyst layer resistance R_CL_ and catalyst layer overpotential *η_CL_* @ 5 A cm^-^² vs. pore size and surface area.


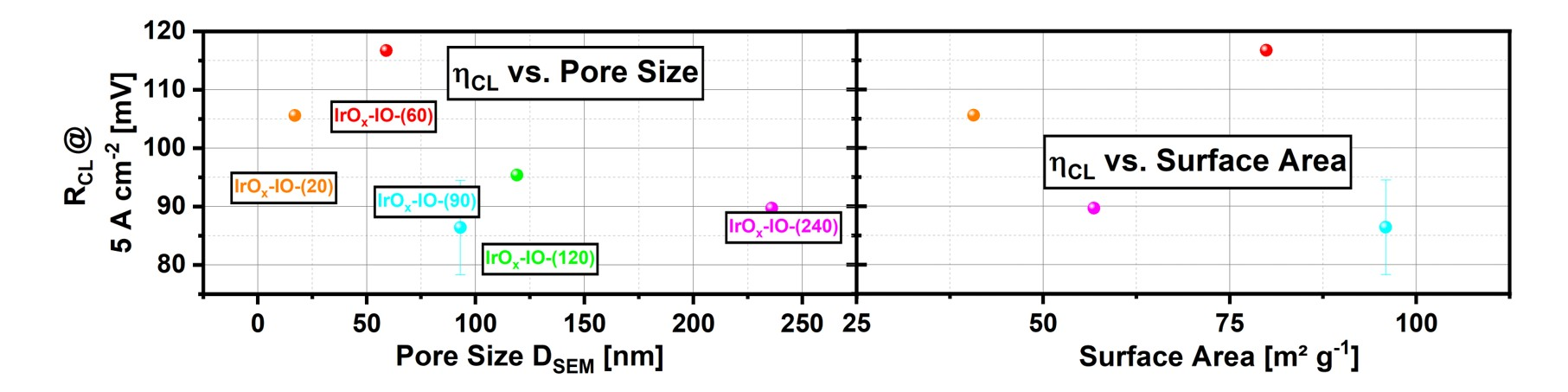


**Figure S19:** Correlation plots of mass transport overpotential *η_mass_* @ 5 A cm^-^² vs. pore size and surface area.


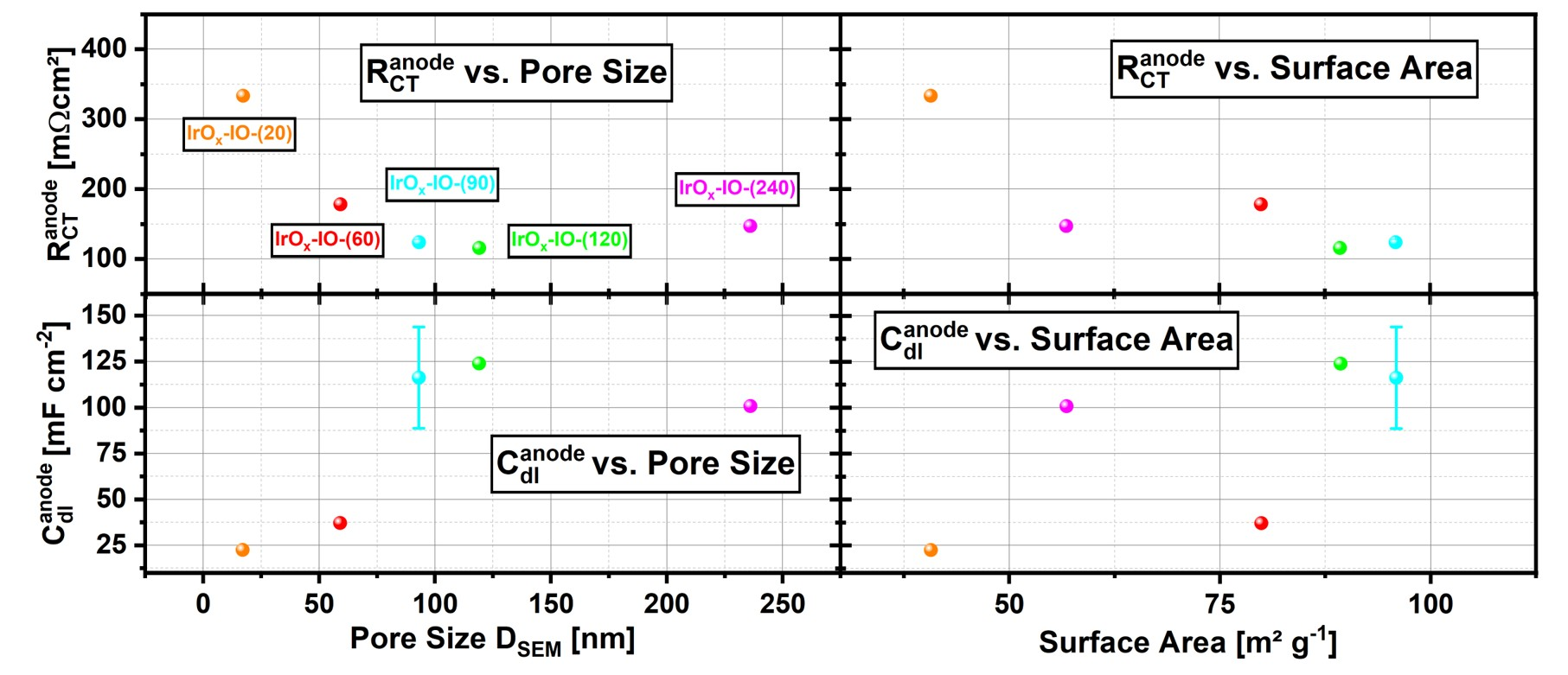


**Figure S20:** Correlation plots of anodic charge transfer resistance and double layer capacitance vs. pore size and surface area.


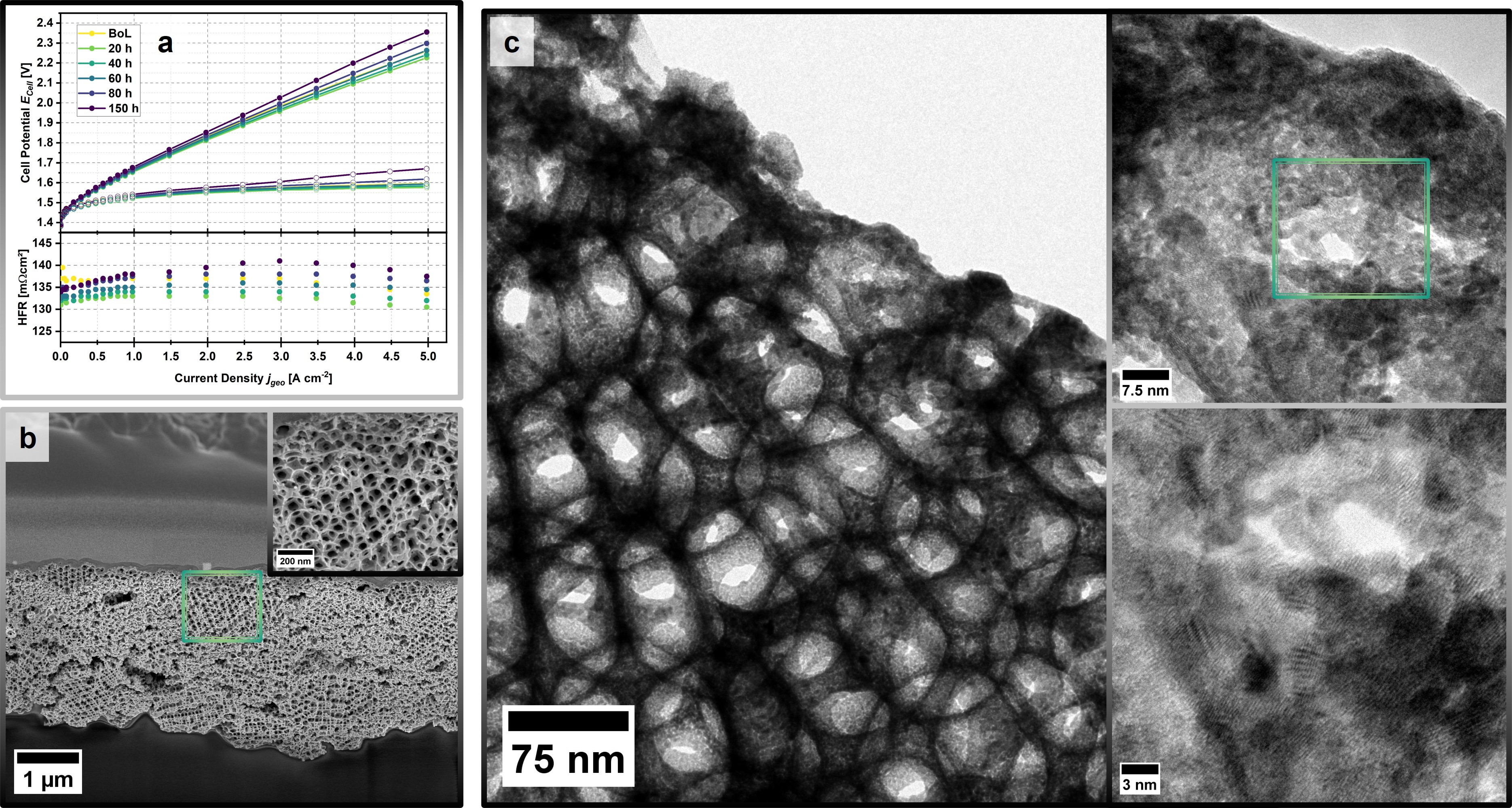


**Figure S21: 150-h stability measurement of IrO_x_-IO-(120):** a: Set of six polarization curves. b: FIB-SEM cross section of the anode catalyst layer. c: TEM micrographs of lamella prepared from b. Test conditions: Both cathode (Umicore Elyst 50, 0.11 mg_Pt_ cm^-^²) and the iridium catalyst (**IrO_x_-IO-(120)**, 0.53 mg cm^-^²) were coated onto the membrane (Nafion NR 115, 5 cm²) using the decal transfer process. Carbon paper (Sigracet 22 BB) was used as the gas diffusion layer on the cathode side, while Pt-sputtered titanium felt was used as porous transport layer on the anode side. The applied torque was 35 Nm. An anode flow of 50 mL/min at 1 atm pressure and 80 °C was used.


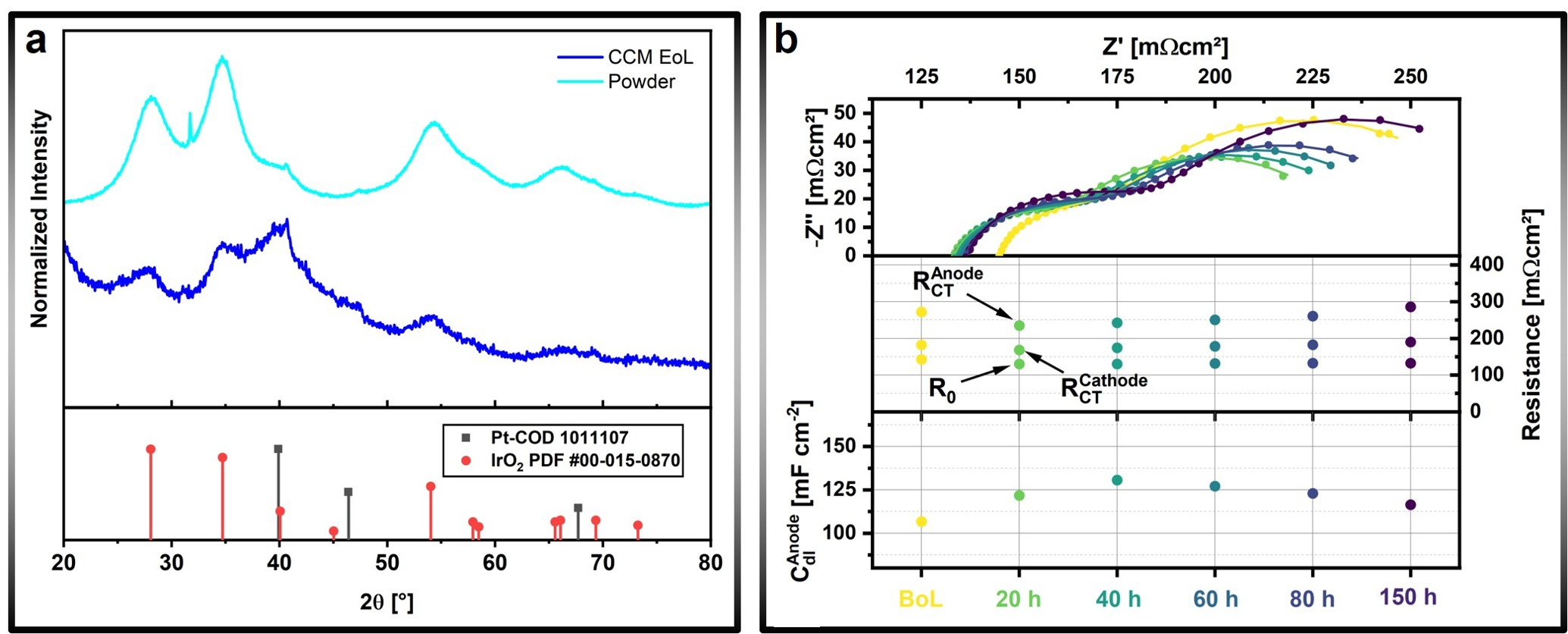
 **Figure S22:** a: X-ray diffractograms of the CCM used for the stability measurement shown in Supplementary Figure 21. b: Top: Impedance spectra recorded at each polarization point shown in Supplementary Figure 21a. Middle: *R_0_*, *R_CT;anode_*, and *R_CT;cathode_* extracted from equivalent circuit modeling. Bottom: Double-layer capacitance obtained from the anodic arc of the corresponding impedance spectra, determined using the same modeling approach.


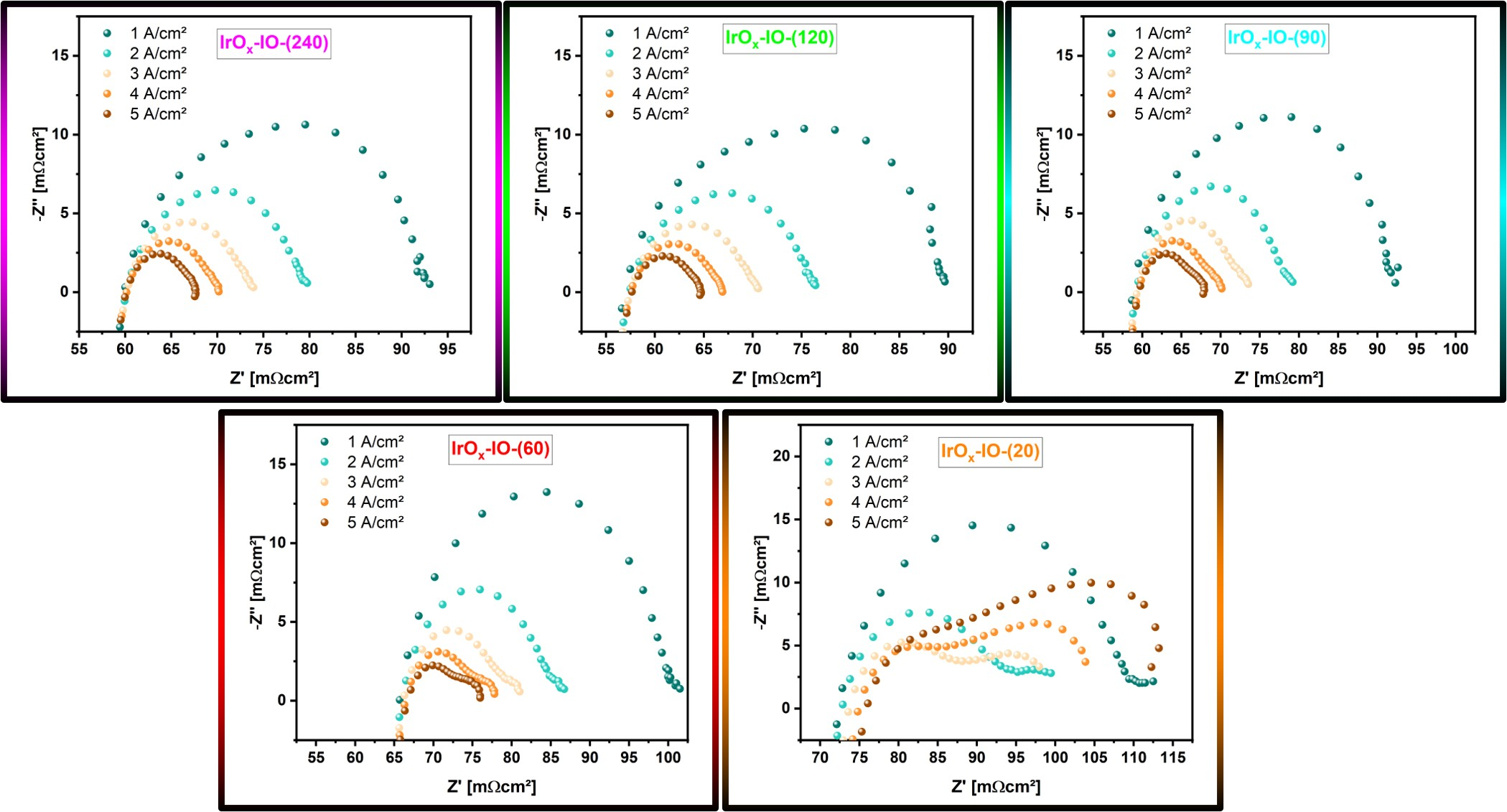
 **Figure S23:** Impedance spectra of the investigated materials at high current densities from 1 to 5 A cm^-^².


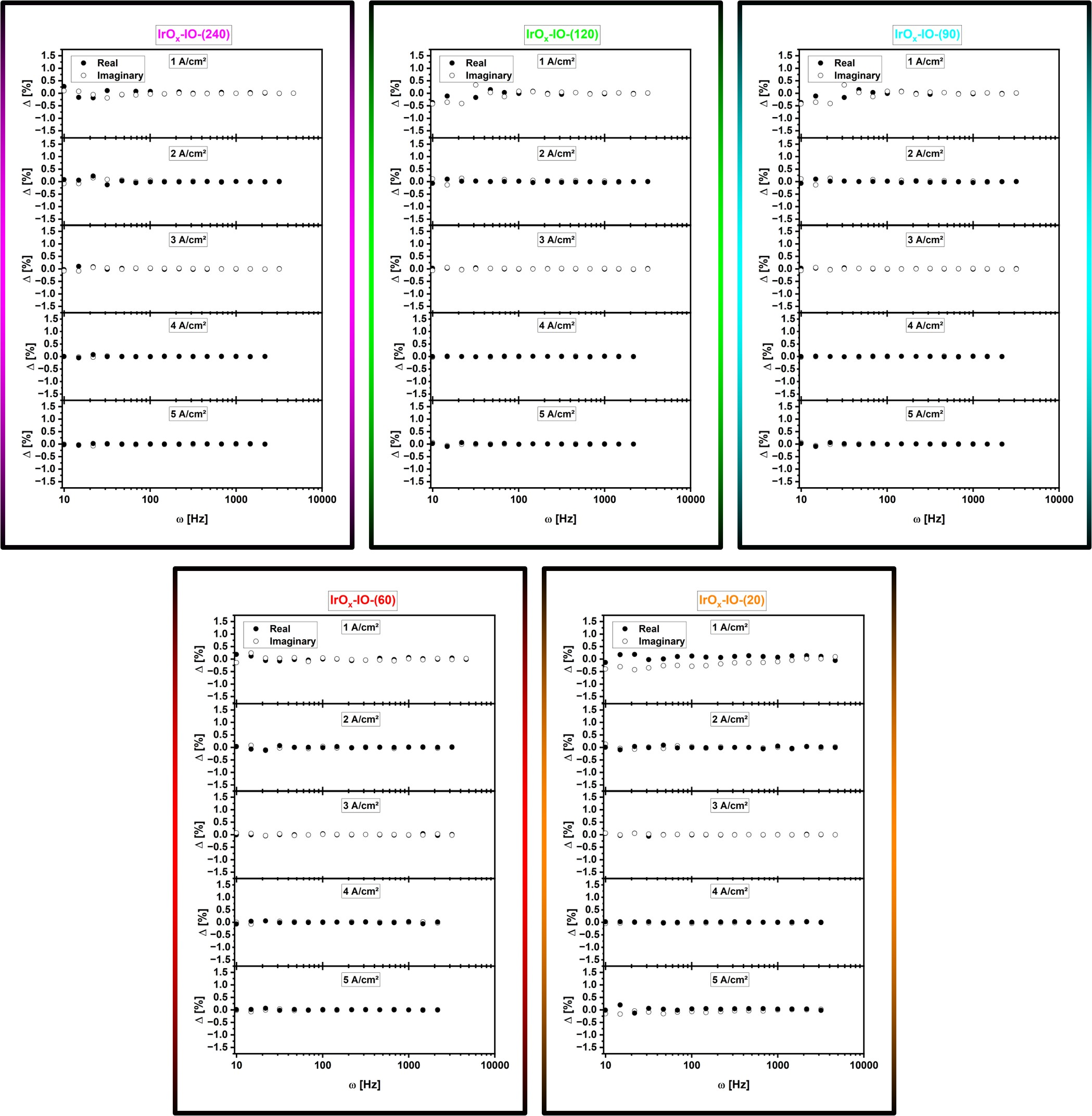


**Figure S24:** Kramers-Kronig validation of the impedance spectra shown in Figure S23.


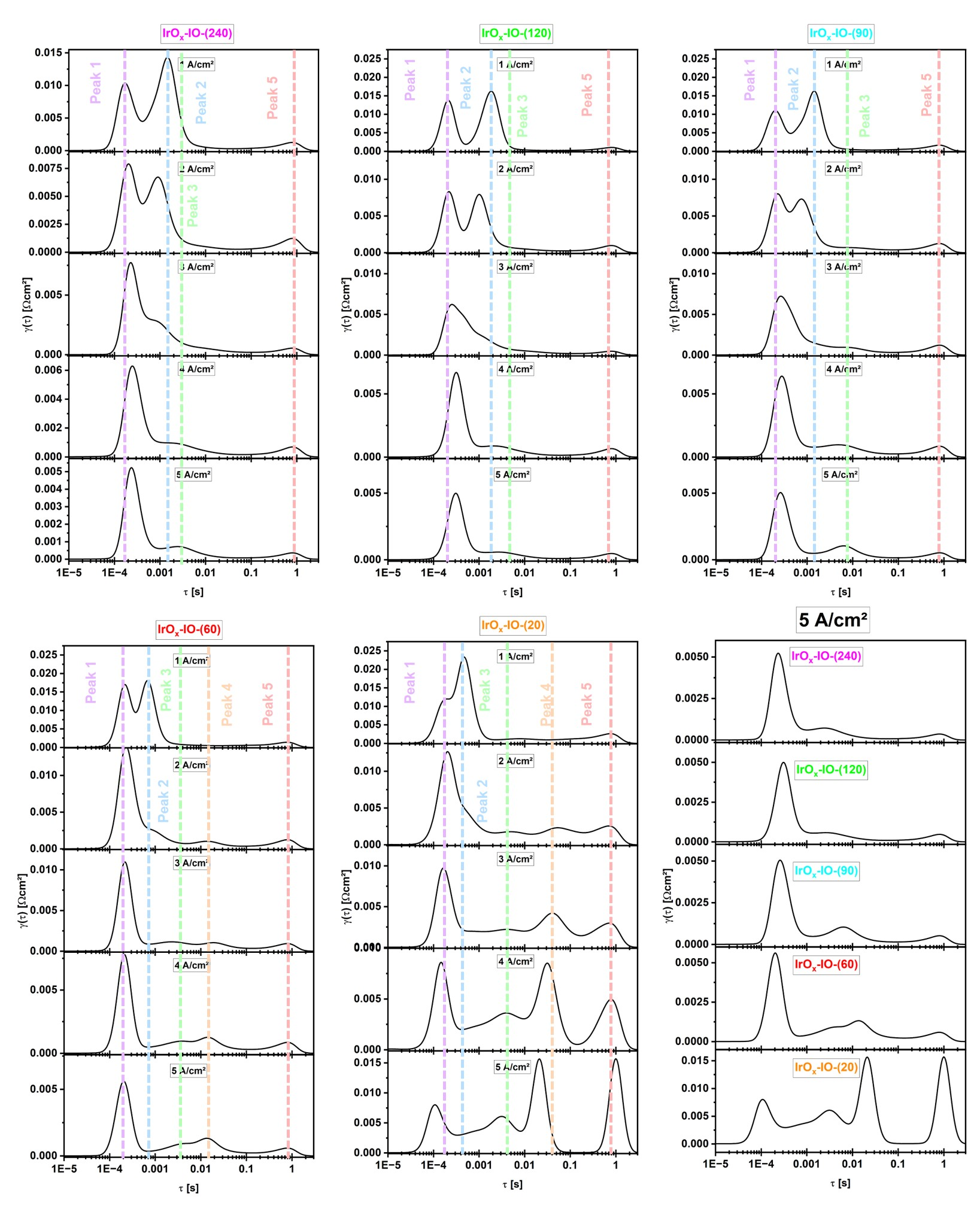


**Figure S25:** Distribution of relaxation times analysis of the investigated materials at current densities from 1 to 5 A cm^-^², with a direct comparison at 5 A cm^-^².


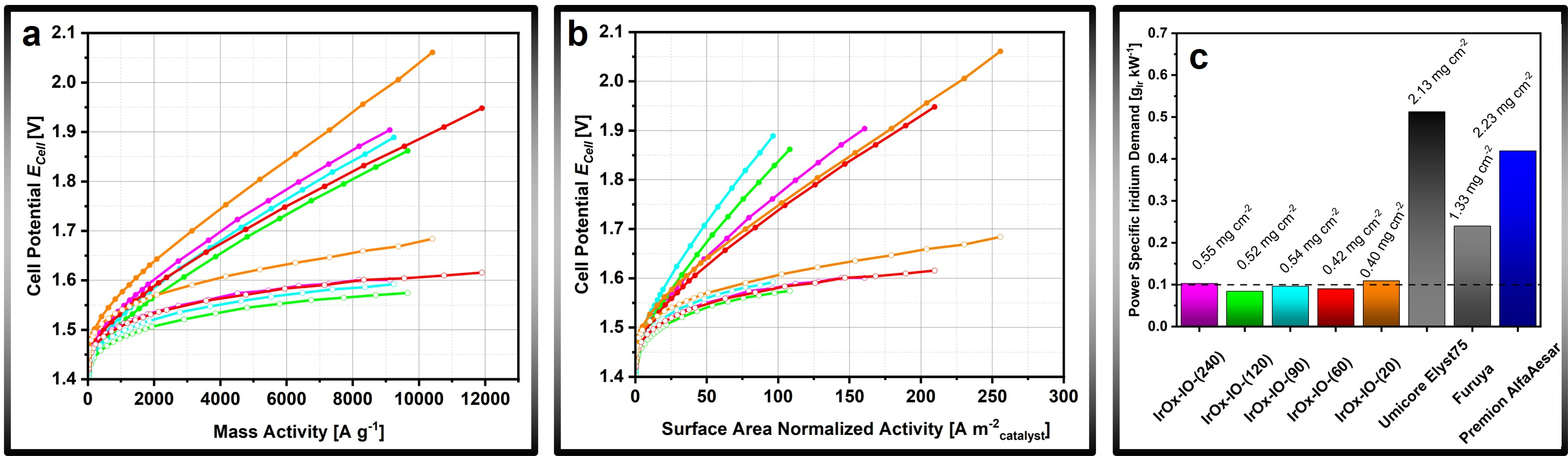
 **Figure S26:** a: Mass normalized activity. b: Surface area normalized activity. c: Mass specific power density at 70% higher heating value.

References

[1] M. Schönleber, D. Klotz, E. Ivers-Tiffée, *Electrochim. Acta* **2014**, *131*, 20.

[2] B. S. Batalla, J. Bachmann, C. Weidlich, *Electrochim. Acta* **2024**, *473*, 143492.

[3] M. Rogler, M. Suermann, R. Wagner, S. Thiele, J. Straub, *J. Electrochem. Soc.* **2023**, *170*, 114521.

[4] M. Maier, K. Smith, J. Dodwell, G. Hinds, P. R. Shearing, D. J. L. Brett, *Int. J. Hydrogen Energy* **2022**, *47*, 30.

[5] T. Schuler, T. J. Schmidt, F. N. Büchi, *J. Electrochem. Soc.* **2019**, *166*, F555.

[6] L. Järvinen, P. Puranen, A. Kosonen, V. Ruuskanen, J. Ahola, P. Kauranen, M. Hehemann, *Int. J. Hydrogen Energy* **2022**, *47*, 31985.

[7] E. Padgett, G. Bender, A. Haug, K. Lewinski, F. Sun, H. Yu, D. A. Cullen, A. J. Steinbach, S. M. Alia, *J. Electrochem. Soc.* **2023**, *170*, 114522.

[8] M. Schalenbach, G. Tjarks, M. Carmo, W. Lueke, M. Mueller, D. Stolten, *J. Electrochem. Soc.* **2016**, *163*, F3197.

[9] T. Srour, K. Kumar, V. Martin, L. Dubau, F. Maillard, B. Gilles, J. Dillet, S. Didierjean, B. Amoury, T. D. Le, G. Maranzana, *Int. J. Hydrogen Energy* **2024**, *58*, 351.

[10] B. Hasa, U. R. Aryal, S. Higashi, N. E. Tolouei, J. T. Lang, B. Erb, A. Smeltz, I. V. Zenyuk, G. Zhu, *Appl. Catal. B* **2025**, *361*, 123456.

[11] M. Suermann, T. J. Schmidt, F. N. Büchi, *Electrochim. Acta* **2018**, *281*, 466.

[12] J. Huang, Y. Gao, J. Luo, S. Wang, C. Li, S. Chen, J. Zhang, *J. Electrochem. Soc.* **2020**, *167*, 037506.

[13] A. Lasia, *J. Electroanal. Chem.* **1995**, *397*, 27.

[14] T. Schuler, R. De Bruycker, T. J. Schmidt, F. N. Büchi, *J. Electrochem. Soc.* **2019**, *166*, F270.

[15] M. Bernt, H. A. Gasteiger, *J. Electrochem. Soc.* **2016**, *163*, F3179.

[16] Y. Jang, C. Seol, S. M. Kim, S. Jang, *Int. J. Hydrogen Energy* **2022**, *47*, 18229.

[17] T. Reier, D. Teschner, T. Lunkenbein, A. Bergmann, S. Selve, R. Kraehnert, R. Schlögl, P. Strasser, *J. Electrochem. Soc.* **2014**, *161*, F876.

[18] D. F. Abbott, D. Lebedev, K. Waltar, M. Povia, M. Nachtegaal, E. Fabbri, C. Copéret, T. J. Schmidt, *Chem. Mater.* **2016**, *28*, 6591.

[19] K. Arunagiri, A. J.-W. Wong, L. Briceno-Mena, H. M. G. H. Elsayed, J. A. Romagnoli, M. J. Janik, C. G. Arges, *Energy Environ. Sci.* **2023**, *16*, 5916.

[20] S. Ott, A. Orfanidi, H. Schmies, B. Anke, H. N. Nong, J. Hubner, U. Gernert, M. Gliech, M. Lerch, P. Strasser, *Nat. Mater.* **2020**, *19*, 77.

[21] S. Siracusano, S. Trocino, N. Briguglio, V. Baglio, A. S. Aricò, *Materials* **2018**, *11*, 1234.

[22] Y. Li, Y. Jiang, J. Dang, X. Deng, B. Liu, J. Ma, F. Yang, M. Ouyang, X. Shen, *Chem. Eng. J.* **2023**, *451*, 138327.

[23] H. Zhang, J. Zhu, J. Xu, C. Wang, H. Yuan, X. Wei, H. Dai, *Appl. Energy* **2025**, *401*, 126609.

[24] A.-L. Chan, H. Yu, K. S. Reeves, S. M. Alia, *J. Power Sources* **2025**, *628*, 235850.
